# Supplementary material for: E4BP4 safeguards brown fat mitochondria from obesity-induced fragmentation via ceramide repression
Source: EMBO Rep. 2026 Jun 22;27(15):4376–411. doi: 10.1038/s44319-026-00826-0 (PMC13458363; doi:10.1038/s44319-026-00826-0)
Supplement: Supplementary file 1 [file 44319_2026_826_MOESM1_ESM.pdf]

Table EV1. Statistical analysis of Oxygen Consumption Rate (OCR) related to Fig. 3H, Fig. 3I, Fig. 5J, Fig. 6G and Fig. 6H.

Table EV1. Statistical analysis of Oxygen Consumption Rate (OCR) related to

	Control		
	BSA	BSA:PAL	pval
NE effect	7.095 ± 2.654	0.87 ± 3.216	*
No mitochondrial oxygen consumption	10.122 ± 1.545	10.971 ± 6.709	n.s.
Basal respiration	33.623 ± 4.109	29.517 ± 5.469	n.s.
Maximal	47.085 ± 7.386	52.634 ± 7.498	n.s.
H+ leak	18.662 ± 5.384	17.119 ± 8.352	n.s.
ATP production	14.961 ± 5.083	12.398 ± 3.071	n.s.

	E4BP4-OE		
	BSA	BSA:PAL	pval
NE effect	4.892 ± 2.751	0.586 ± 2.332	0,054
No mitochondrial oxygen consumption	12.736 ± 0.706	11.849 ± 1.724	n.s.
Basal respiration	35.011 ± 0.739	31.142 ± 5.574	n.s.
Maximal	64.605 ± 5.169	49.608 ± 2.515	**
H+ leak	13.557 ± 4.637	18.486 ± 4.264	n.s.
ATP production	21.453 ± 4.287	12.655 ± 3.211	n.s.

	BSA:PAL		
	Control	E4BP4-OE	pval
NE effect	0.87 ± 3.216	0.586 ± 2.332	n.s.
No mitochondrial oxygen consumption	10.971 ± 6.709	11.849 ± 1.724	n.s.
Basal respiration	29.517 ± 5.469	31.142 ± 5.574	n.s.
Maximal	52.634 ± 7.498	49.608 ± 2.515	n.s.
H+ leak	17.119 ± 8.352	18.486 ± 4.264	n.s.
ATP production	12.398 ± 3.071	12.655 ± 3.211	n.s.

Figure 3H

24 hours BSA or BSA:PAL (E4BP4-OE)

	Control		
	BSA	BSA:PAL	pval
NE effect	10.837 ± 2.801	0.011 ± 1.104	**
No mitochondrial oxygen consumption	9.251 ± 2.975	12.091 ± 2.574	n.s.
Basal respiration	22.403 ± 8.647	4.024 ± 4.583	*
Maximal	34.313 ± 3.842	1.385 ± 2.749	***
H+ leak	6.509 ± 4.555	3.395 ± 5.409	n.s.
ATP production	15.894 ± 8.215	0.629 ± 2.258	**

	E4BP4-OE		
	BSA	BSA:PAL	pval
NE effect	9.889 ± 1.981	4.379 ± 1.057	**
No mitochondrial oxygen consumption	11.216 ± 3.966	14.733 ± 3.986	n.s.
Basal respiration	15.324 ± 5.712	12.532 ± 5.346	n.s.
Maximal	45.211 ± 4.601	24.078 ± 4.152	**
H+ leak	7.474 ± 3.801	6.903 ± 6.922	n.s.
ATP production	7.85 ± 4.192	5.629 ± 2.032	n.s.

	BSA:PAL		
	Control	E4BP4-OE	pval
NE effect	0.011 ± 1.104	4.379 ± 1.057	**
No mitochondrial oxygen consumption	12.091 ± 2.574	14.733 ± 3.986	n.s.
Basal respiration	4.024 ± 4.583	12.532 ± 5.346	**
Maximal	1.385 ± 2.749	24.078 ± 4.152	***
H+ leak	3.395 ± 5.409	6.903 ± 6.922	n.s.
ATP production	0.629 ± 2.258	5.629 ± 2.032	*

Figure 3I

	BSA:PAL		
	**E4BP4**	**E4BP4+CERS6**	Pval
NE effect	4.922 ± 1.438	1.109 ± 0.601	**
No mitochondrial oxygen consumption	14.303 ± 3.548	11.509 ± 3.201	n.s.
Basal respiration	28.976 ± 1.983	23.391 ± 2.238	*
Maximal	64.219 ± 7.942	34.646 ± 4.202	***
H+ leak	13.739 ± 2.031	15.661 ± 2.951	n.s
ATP production	15.237 ± 2.412	7.729 ± 2.215	**

Figure 5J

6 hours BSA or BSA:PAL (Enhancer)

	Non-edited		
	BSA	BSA:PAL	pval
NE effect	9.565 ± 3.618	0.008 ± 0.1	***
No mitochondrial oxygen consumption	13.407 ± 5.783	11.906 ± 2.142	n.s.
Basal respiration	41.629 ± 7.314	36.239 ± 9.022	n.s.
Maximal	86.666 ± 7.076	65.062 ± 7.623	**
H+ leak	21.973 ± 6.677	19.649 ± 1.188	n.s.
ATP production	19.655 ± 5.984	16.589 ± 8.806	n.s.

	ΔE		
	BSA	BSA:PAL	pval
NE effect	16.115 ± 6.206	2.99 ± 0.643	**
No mitochondrial oxygen consumption	16.734 ± 3.978	15.83 ± 3.997	n.s.
Basal respiration	30.139 ± 11.436	32.362 ± 10.374	n.s.
Maximal	87.857 ± 6.252	52.706 ± 8.719	**
H+ leak	18.174 ± 10.47	15.729 ± 7.452	n.s.
ATP production	11.966 ± 6.105	16.633 ± 5.423	n.s.

	BSA:PAL		
	Non-edited	ΔE	pval
NE effect	0.008 ± 0.1	2.99 ± 0.643	**
No mitochondrial oxygen consumption	11.906 ± 2.142	15.83 ± 3.997	n.s
Basal respiration	36.239 ± 9.022	32.362 ± 10.374	n.s
Maximal	65.062 ± 7.623	52.706 ± 8.719	*
H+ leak	19.649 ± 1.188	15.729 ± 7.452	n.s.
ATP production	16.589 ± 8.806	16.633 ± 5.423	n.s.

Figure 6G

24 hours BSA or BSA:PAL (Enhancer)

	Non-edited		
	BSA	BSA:PAL	pval
NE effect	8.229 ± 1.043	0.011 ± 1.104	***
No mitochondrial oxygen consumption	10.405 ± 7.887	7.594 ± 1.355	n.s.
Basal respiration	29.072 ± 7.969	12.48 ± 2.818	***
Maximal	61.99 ± 8.286	7.905 ± 1.801	***
H+ leak	13.004 ± 10.134	6.578 ± 1.275	n.s.
ATP production	16.068 ± 1.683	5.902 ± 2.738	**

	ΔE		
	BSA	BSA:PAL	pval
NE effect	7.66 ± 0.781	0.277 ± 1.272	***
No mitochondrial oxygen consumption	9.934 ± 7.431	12.213 ± 4.054	n.s.
Basal respiration	27.949 ± 7.453	24.172 ± 5.346	n.s.
Maximal	59.843 ± 7.503	43.366 ± 7.521	**
H+ leak	12.503 ± 9.463	16.087 ± 4.114	n.s.
ATP production	15.446 ± 4.206	8.085 ± 4.209	*

	BSA:PAL		
	Non-edited	ΔE	pval
NE effect	0.011 ± 1.104	0.277 ± 1.272	n.s.
No mitochondrial oxygen consumption	7.594 ± 1.355	12.213 ± 4.054	n.s.
Basal respiration	12.48 ± 2.818	24.172 ± 5.346	**
Maximal	7.905 ± 1.801	43.366 ± 7.521	***
H+ leak	6.578 ± 1.275	16.087 ± 4.114	**
ATP production	5.902 ± 2.738	8.085 ± 4.209	n.s.

Figure 6H
