# Supplementary material for: E4BP4 safeguards brown fat mitochondria from obesity-induced fragmentation via ceramide repression
Source: EMBO Rep. 2026 Jun 22;27(15):4376–411. doi: 10.1038/s44319-026-00826-0 (PMC13458363; doi:10.1038/s44319-026-00826-0)
Supplement: Supplementary file 2 [file 44319_2026_826_MOESM21_ESM.pdf]

Expanded View Figures

Figure EV1. Analysis of E4BP4-OE in DIO mice using AAV constitutive CMV promoter (Related to Fig. 2).

(**A**) Schematic illustration of experimental design (**A–H**) for overexpression of E4bp4 (E4BP4-OE) or control in iBAT. The cartoon was created using Biorender. (**B**) Body weight of Control or E4BP4-OE mice under HFD at 28–29 °C. $n = 5$ per group. Data were presented as mean ± SEM. Statistical significance was determined by two-way ANOVA followed by Tukey's post hoc test. The p value is displayed when $p < 0.05$. (**C**) Cumulative food intake of Control or E4BP4-OE mice under HFD at 28–29 °C. $n = 5$ per group. Data were presented as mean ± SEM. Statistical significance was determined by two-way ANOVA followed by Tukey's post hoc test. The p value is displayed when $p < 0.05$. (**D**) iBAT temperature of Control or E4BP4-OE mice under HFD at 28–29 °C. $n = 5$ per group. Data were presented as mean ± SEM. Statistical significance was determined by two-way ANOVA followed by Tukey's post hoc test. (**E**) Relative E4bp4 mRNA levels in iBAT of Control and E4BP4-OE mice fed a HFD at 28–29 °C. $n = 5$ per group. Data were presented as mean ± SEM. Two-sided P values were calculated using an unpaired Student's t-test. Control vs E4BP4-OE: $p < 0.0001$. (**F**) Tissue weight normalized to body weight of Control and E4BP4-OE mice fed a HFD at 28–29 °C. $n = 5$ per group. Data were presented as mean ± SEM. Two-sided P values were calculated using an unpaired Student's t-test. Control vs E4BP4-OE: $p = 0.0481$. (**G**) Left: representative images of Hematoxylin and eosin (H&E) staining of iBAT from control and E4BP4-OE mice fed a HFD at 28–29 °C. Scale bar: 50 µm. Right: Lipid droplet area quantification. $n = 3$ per group. Data were presented as mean ± SEM. Two-sided P values were calculated using an unpaired Student's t-test. Control vs E4BP4-OE: $p = 0.0010$. (**H**) Images of mitochondrial iBAT of Control and E4BP4-OE by TOM20 immunofluorescence analysis. Representative image. Scale bar, 50 µm. Source data are available online for this figure.

© The Author(s)

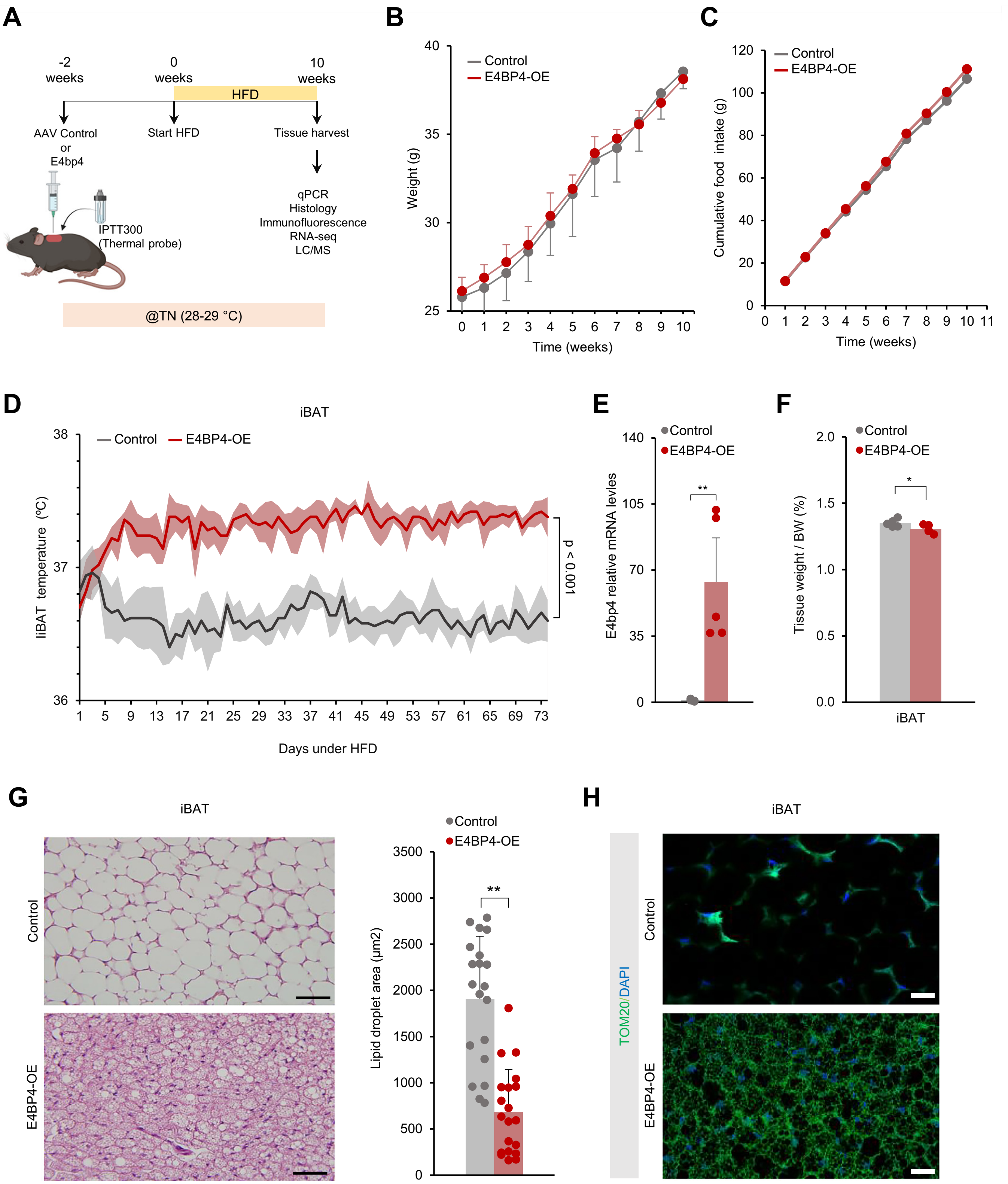
A
-2 weeks
0 weeks
10 weeks
HFD
AAV Control or E4bp4
Start HFD
Tissue harvest
qPCR
Histology
Immunofluorescence
RNA-seq
LC/MS
IPTT300 (Thermal probe)
@TN (28-29 °C)
B
Control
E4BP4-OE
Weight (g)
Time (weeks)
C
Control
E4BP4-OE
Cumulative food intake (g)
Time (weeks)
D
iBAT
Control
E4BP4-OE
iBAT temperature (°C)
p < 0.001
Days under HFD
E
Control
E4BP4-OE
E4bp4 relative mRNA levles
**
F
Control
E4BP4-OE
Tissue weight / BW (%)
*
iBAT
G
iBAT
Control
E4BP4-OE
Control
E4BP4-OE
Lipid droplet area (μm2)
**
H
iBAT
TOM20/DAPI
Control
E4BP4-OE

 © The Author(s)

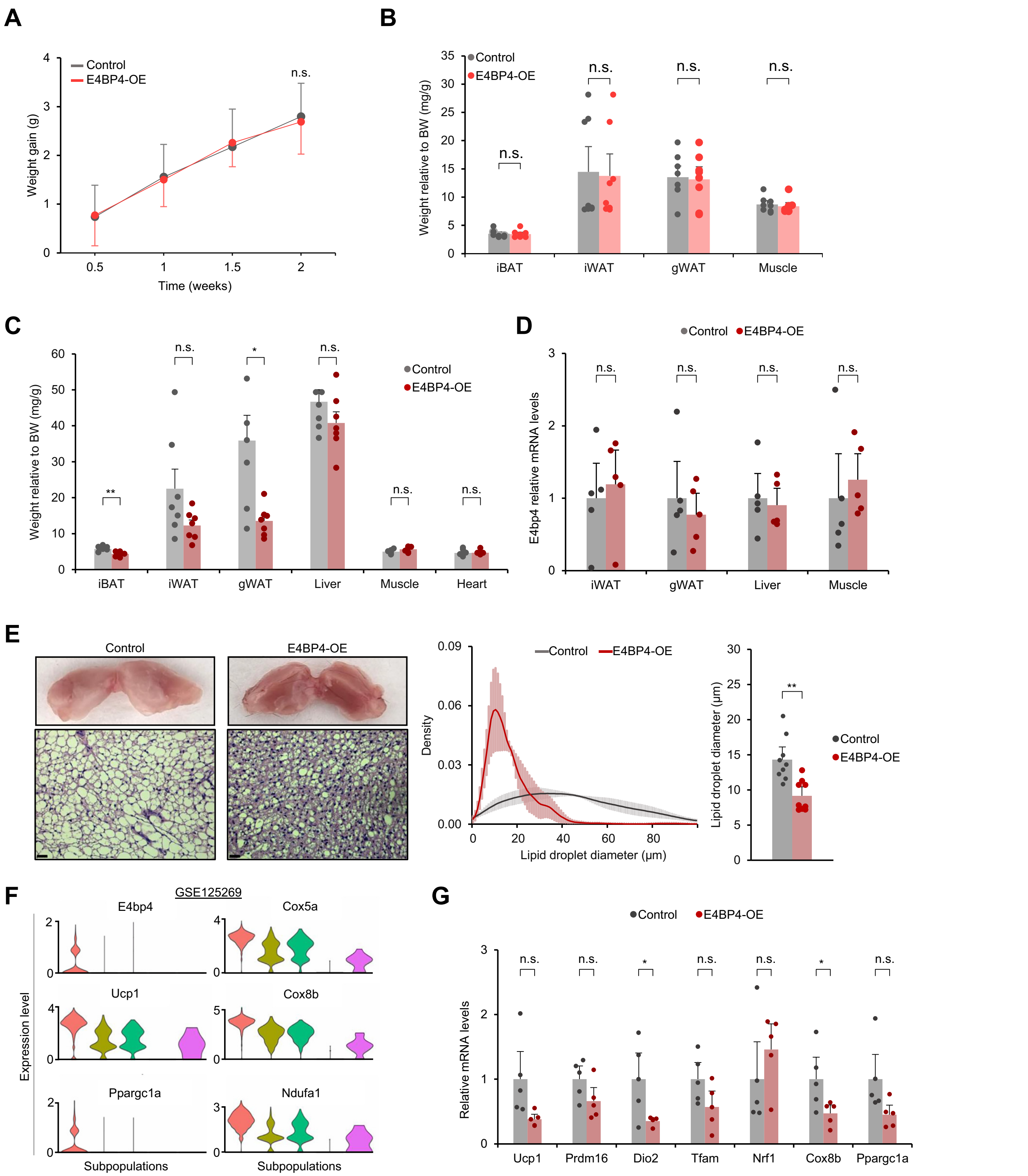
A
Control
E4BP4-OE
n.s.
Weight gain (g)
Time (weeks)
B
Control
E4BP4-OE
n.s.
Weight relative to BW (mg/g)
iBAT
iWAT
gWAT
Muscle
C
Control
E4BP4-OE
n.s.
*
**
Weight relative to BW (mg/g)
iBAT
iWAT
gWAT
Liver
Muscle
Heart
D
Control
E4BP4-OE
n.s.
E4bp4 relative mRNA levels
iWAT
gWAT
Liver
Muscle
E
Control
E4BP4-OE
Density
Lipid droplet diameter (µm)
**
F
GSE125269
E4bp4
Cox5a
Ucp1
Cox8b
Ppargc1a
Ndufa1
Expression level
Subpopulations
G
Control
E4BP4-OE
n.s.
*
Relative mRNA levels
Ucp1
Prdm16
Dio2
Tfam
Nrf1
Cox8b
Ppargc1a

Figure EV2. Analysis of E4BP4-OE in DIO mice using AAV adiponectin promoter (Related to Fig. 2).

(**A**) Weight of E4BP4-OE or control mice under HFD at 28–29 °C during 2 weeks. $n = 8$ per group. Data were presented as mean ± SEM. Statistical significance was determined by two-way ANOVA followed by Tukey's post hoc test. The p value at 2 weeks of Control vs E4BP4-OE: $p = 0.6591$. ns not significant. (**B**) Tissue weight normalized to body weight of Control and E4BP4-OE mice fed a HFD at 28–29 °C during 2 weeks. $n = 8$ per group. Data were presented as mean ± SEM. Two-sided P values were calculated using an unpaired Student's t-test. P values for each comparison: iBAT of Control vs E4BP4-OE: $p = 0.8431$; iWAT of Control vs E4BP4-OE: $p = 0.8672$; gWAT of Control vs E4BP4-OE: $p = 0.8591$; Muscle of Control vs E4BP4-OE: $p = 0.6282$. ns not significant. (**C**) Tissue weight normalized to body weight of Control and E4BP4-OE mice fed a HFD at 28–29 °C during 10 weeks. $n = 7$ per group. Data were presented as mean ± SEM. Two-sided P values were calculated using an unpaired Student's t-test. P values for each comparison: iBAT of Control vs E4BP4-OE: $p = 0.0037$; iWAT of Control vs E4BP4-OE: $p = 0.0963$; gWAT of Control vs E4BP4-OE: $p = 0.0110$; Liver of Control vs E4BP4-OE: $p = 0.2234$; Muscle of Control vs E4BP4-OE: $p = 0.0823$; Heart of Control vs E4BP4-OE: $p = 0.8921$. ns not significant. (**D**) Relative E4bp4 mRNA levels in the indicated tissues of Control and E4BP4-OE mice fed a HFD at 28–29 °C during 10 weeks. $n = 7$ per group. Data were presented as mean ± SEM. Two-sided P values were calculated using an unpaired Student's t-test. P values for each comparison: iWAT of Control vs E4BP4-OE: $p = 0.6630$; gWAT of Control vs E4BP4-OE: $p = 0.5597$; Liver of Control vs E4BP4-OE: $p = 0.7256$; Muscle of Control vs E4BP4-OE: $p = 0.5873$. ns not significant. (**E**) Left: representative images of Hematoxylin and eosin (H&E) staining of iBAT from control and E4BP4-OE mice fed a HFD at 28–29 °C during 10 weeks. Scale bar: 50 µm. Right: lipid droplet diameter distribution and quantification. $n = 3$ per group. Data were presented as mean ± SEM. Two-sided P values were calculated using an unpaired Student's t-test. Control vs E4BP4-OE: $p = 0.0010$. (**F**) Violin plots showing the distribution of normalized expression values of the indicated genes across cells of the adipocyte clusters. $n = 3602$ primary brown adipocytes. (**G**) Relative mRNA levels of indicated genes of Control and E4BP4-OE mice fed a HFD at 28–29 °C during 10 weeks. $n = 7$ per group. Data were presented as mean ± SEM. Two-sided P values were calculated using an unpaired Student's t-test. P values for each comparison: Ucp1 of Control vs E4BP4-OE: $p = 0.0548$; Prdm16 of Control vs E4BP4-OE: $p = 0.1051$; Dio2 of Control vs E4BP4-OE: $p = 0.0373$; Tfam of Control vs E4BP4-OE: $p = 0.0943$; Nrf1 of Control vs E4BP4-OE: $p = 0.3307$; Cox8b of Control vs E4BP4-OE: $p = 0.0481$; Pppargc1a of Control vs E4BP4-OE: $p = 0.0687$. ns not significant. Source data are available online for this figure.

 © The Author(s)

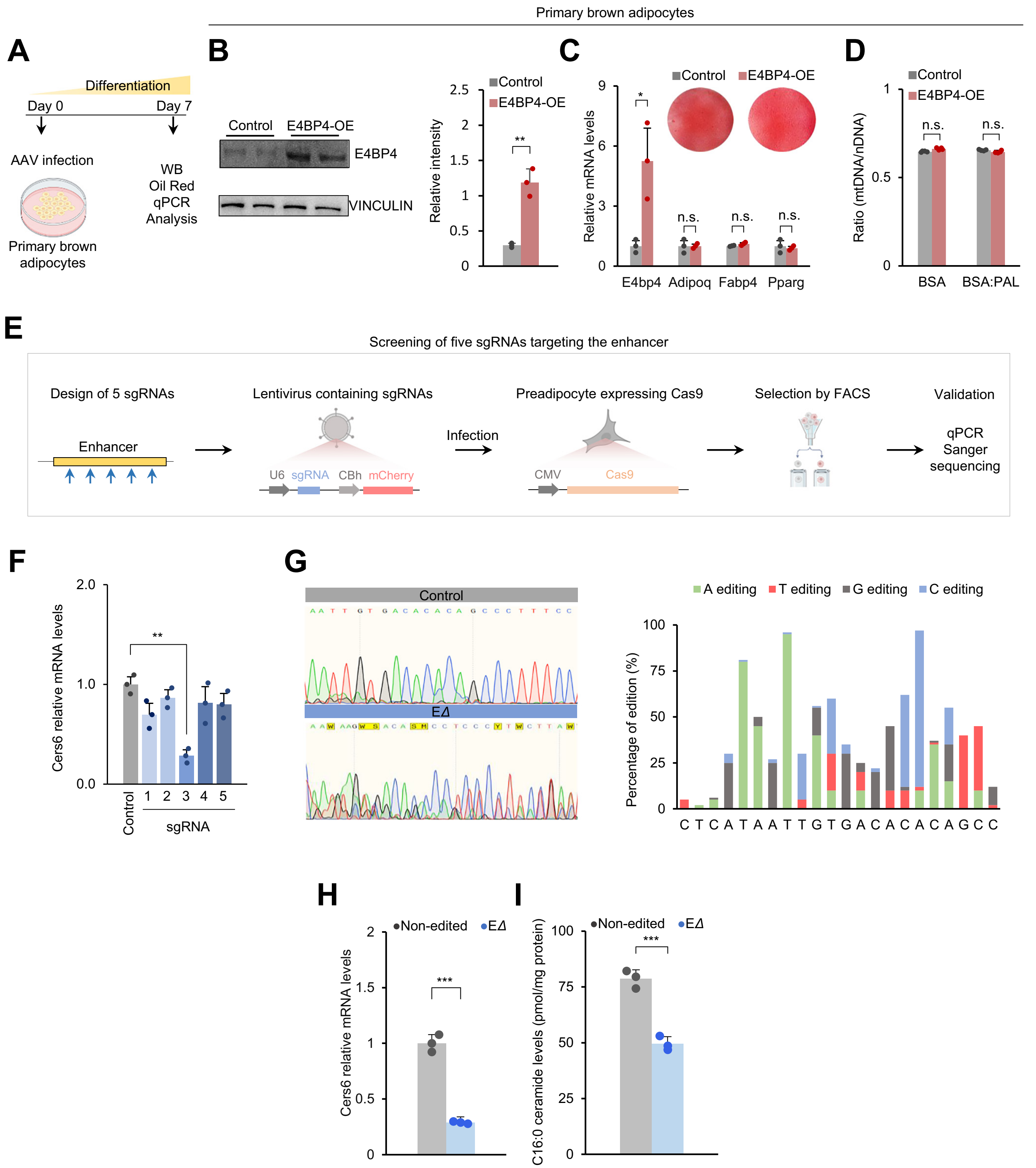
Primary brown adipocytes
A
Differentiation
Day 0
Day 7
AAV infection
WB
Oil Red
qPCR
Analysis
Primary brown adipocytes
B
Control
E4BP4-OE
E4BP4
VINCULIN
Control
E4BP4-OE
Relative intensity
**
C
Control
E4BP4-OE
Relative mRNA levels
*
n.s.
E4bp4
Adipoq
Fabp4
Pparg
D
Control
E4BP4-OE
Ratio (mtDNA/nDNA)
n.s.
BSA
BSA:PAL
E
Screening of five sgRNAs targeting the enhancer
Design of 5 sgRNAs
Enhancer
Lentivirus containing sgRNAs
U6
sgRNA
CBh
mCherry
Infection
Preadipocyte expressing Cas9
CMV
Cas9
Selection by FACS
Validation
qPCR
Sanger
sequencing
F
Cers6 relative mRNA levels
**
Control
sgRNA
G
Control
EΔ
A editing
T editing
G editing
C editing
Percentage of edition (%)
C T C A T A A T T G T G A C A C A C A G C C
H
Non-edited
EΔ
Cers6 relative mRNA levels
***
I
Non-edited
EΔ
C16:0 ceramide levels (pmol/mg protein)
***

Figure EV3. E4bp4 gain-of-function and mutant (ΔE) Cers6 enhancer (E0697815) models in brown adipocytes (Related to Figs. 3 and 5).

(**A**) Schematic illustration of experimental design (B-D) for E4bp4 overexpression (E4BP4-OE) or Control in primary brown adipocytes. The cartoon was created using Biorender. (**B**) Left: Western blotting for E4bp4 in primary brown adipocytes of Control and E4BP4-OE. Representative results are shown from two independent experiments. The gel source data are presented in the Source data. Right: quantification of Western blot. $n = 2$ independent experiments. Data were presented as mean ± SEM. Two-sided *P* values were calculated using an unpaired Student's *t*-test. Control vs E4BP4-OE: $p = 0.0015$. (**C**) Relative mRNA levels of indicated genes in primary brown adipocytes of the Control and E4BP4-OE groups. $n = 3$ per group. Data were presented as mean ± SEM. Two-sided *P* values were calculated using an unpaired Student's *t*-test. *P* values for each comparison: E4bp4 of Control vs E4BP4-OE: $p = 0.0227$; Adipoq of Control vs E4BP4-OE: $p = 0.2801$; Fabp4 of Control vs E4BP4-OE: $p = 0.0595$; Pparg of Control vs E4BP4-OE: $p = 0.2852$. (**D**) Ratio of mitochondrial DNA (mtDNA) and nuclear DNA (nDNA) in primary brown adipocytes of the Control and E4BP4-OE groups. $n = 3$ per group. Data were presented as mean ± SEM. *P* values for each comparison: BSA of Control vs E4BP4-OE: $p = 0.0576$; BSA:PAL of Control vs E4BP4-OE: $p = 0.1106$. ns not significant. (**E**) Schematic illustration of experimental design for the screening of sgRNAs targeting the Cers6 E0697815 enhancer (**F**, **G**). The cartoon was created using Biorender. (**F**) Relative Cers6 mRNA levels in Cers6 mutant E0697815 enhancer (ΔE) brown adipocytes for the validation of sgRNA. $n = 3$ per group. Data were expressed as mean ± SEM. Statistical significance was determined by one-way ANOVA followed by Dunnett's multiple comparisons test to compare each sgRNA treatment group against the control group. *P* values for each comparison: Control vs sgRNA1: $p = 0.0351$; Control vs sgRNA2: $p = 0.1630$; Control vs sgRNA3: $p = 0.0011$; Control vs sgRNA4: $p = 0.2194$; Control vs sgRNA5: $p = 0.1028$. (**G**) Left: Sequencing analysis of CRISPR/Cas9-mediated Cers6 E0697815 enhancer (ΔE) ablation in brown adipocytes. Nucleotide sequences of the targeted enhancer in cells treated with sgRNA and control. Right: percentage of nucleotide mutations derived from Sanger sequencing data. (**H**) Relative Cers6 mRNA levels in Cers6 E0697815 mutant enhancer (ΔE) brown adipocytes or Control. $n = 3$ per group. Data were presented as mean ± SEM. Two-sided *P* values were calculated using an unpaired Student's *t*-test. Nonedited vs EΔ: $p = 0.0001$. (**I**) C16:0 ceramide quantification by mass spectrometry in Cers6 mutant E0697815 enhancer (ΔE) brown adipocytes or Control. $n = 3$ per group. Data were presented as mean ± SEM. Two-sided *P* values were calculated using an unpaired Student's *t*-test. Nonedited vs EΔ: $p = 0.0005$. Source data are available online for this figure.

 © The Author(s)

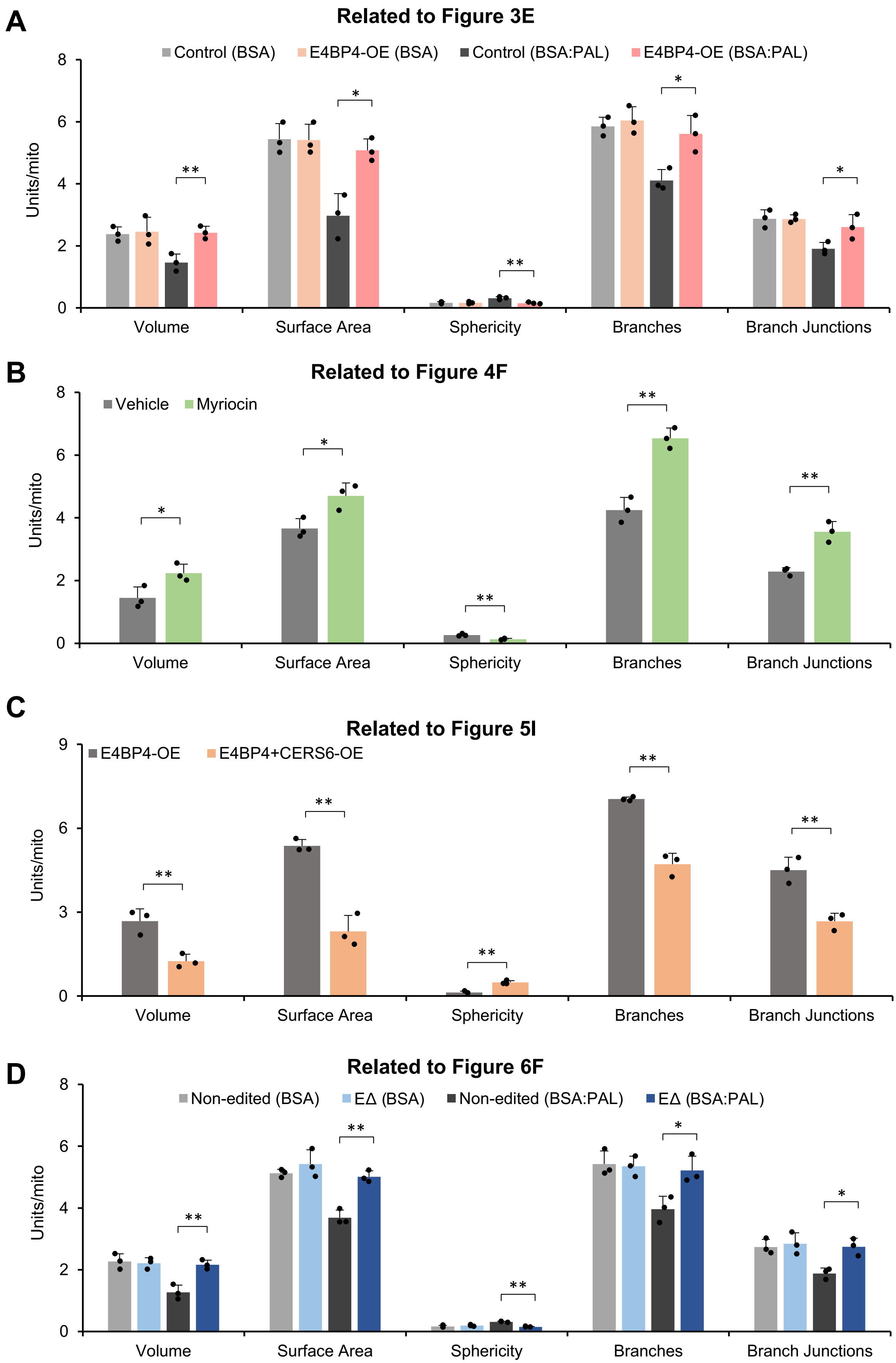
A
Related to Figure 3E
Control (BSA)
E4BP4-OE (BSA)
Control (BSA:PAL)
E4BP4-OE (BSA:PAL)
Units/mito
Volume
Surface Area
Sphericity
Branches
Branch Junctions
B
Related to Figure 4F
Vehicle
Myriocin
Units/mito
Volume
Surface Area
Sphericity
Branches
Branch Junctions
C
Related to Figure 5I
E4BP4-OE
E4BP4+CERS6-OE
Units/mito
Volume
Surface Area
Sphericity
Branches
Branch Junctions
D
Related to Figure 6F
Non-edited (BSA)
EΔ (BSA)
Non-edited (BSA:PAL)
EΔ (BSA:PAL)
Units/mito
Volume
Surface Area
Sphericity
Branches
Branch Junctions

© The Author(s)

Figure EV4. Mitochondrial morphology characterization in brown adipocytes.

(**A**) Quantification of mitochondrial morphology. Mean mitochondrial volume (units in μm^3), mean mitochondrial surface area (units in μm^2), mitochondrial sphericity index, mean number of branches, and junctions per mitochondrion. Related to Fig. 3E. Data were presented as mean ± SEM. $n = 3$ per group. Statistical significance was determined by two-way ANOVA followed by Tukey's post hoc test. The p value is displayed when $p < 0.05$. P values for each comparison: volume of Control (BSA:PAL) vs E4BP4-OE (BSA:PAL): $p = 0.0086$; Surface area of Control (BSA:PAL) vs E4BP4-OE (BSA:PAL): $p = 0.0103$; Sphericity of Control (BSA:PAL) vs E4BP4-OE (BSA:PAL): $p = 0.0077$; Branches of Control (BSA:PAL) vs E4BP4-OE (BSA:PAL): $p = 0.0193$; Branch junctions of Control (BSA:PAL) vs E4BP4-OE (BSA:PAL): $p = 0.0481$. (**B**) Quantification of mitochondrial morphology. Mean mitochondrial volume (units in μm^3), mean mitochondrial surface area (units in μm^2), mitochondrial sphericity index, mean number of branches and junctions per mitochondrion. Related to Fig. 4F. Data were presented as mean ± SEM. $n = 3$ per group. Two-sided P values were calculated using an unpaired Student's t-test. The p value is displayed when $p < 0.05$. P values for each comparison: volume of Vehicle vs Myriocin: $p = 0.0382$; Surface area of Vehicle vs Myriocin: $p = 0.0251$; Sphericity of Vehicle vs Myriocin: $p = 0.0087$; Branches of Vehicle vs Myriocin: $p = 0.0015$; Branch Junctions of Vehicle vs Myriocin: $p = 0.0032$. (**C**) Quantification of mitochondrial morphology. Mean mitochondrial volume (units in μm^3), mean mitochondrial surface area (units in μm^2), mitochondrial sphericity index, mean number of branches and junctions per mitochondrion. Related to Fig. 5I. Data were presented as mean ± SEM. $n = 3$ per group. Two-sided P values were calculated using an unpaired Student's t-test. The p value is displayed when $p < 0.05$. P values for each comparison: volume of E4BP4-OE vs E4BP4 + CERS6-OE: $p = 0.0078$; Surface area of E4BP4-OE vs E4BP4 + CERS6-OE: $p = 0.0010$; Sphericity of E4BP4-OE vs E4BP4 + CERS6-OE: $p = 0.0015$; Branches of E4BP4-OE vs E4BP4 + CERS6-OE: $p = 0.001$; Branch Junctions of E4BP4-OE vs E4BP4 + CERS6-OE: $p = 0.0045$. (**D**) Quantification of mitochondrial morphology. Mean mitochondrial volume (units in μm^3), mean mitochondrial surface area (units in μm^2), mitochondrial sphericity index, mean number of branches and junctions per mitochondrion. Related to Fig. 6F. Data were presented as mean ± SEM. $n = 3$ per group. Statistical significance was determined by two-way ANOVA followed by Tukey's post hoc test. The p value is displayed when $p < 0.05$. P values for each comparison: volume of nonedited(BSA:PAL) vs EΔ(BSA:PAL): $p = 0.0053$; Surface area of nonedited(BSA:PAL) vs EΔ(BSA:PAL): $p = 0.0018$; Sphericity of nonedited(BSA:PAL) vs EΔ(BSA:PAL): $p = 0.0012$; Branches of nonedited(BSA:PAL) vs EΔ(BSA:PAL): $p = 0.0247$; Branch junctions of Nonedited(BSA:PAL) vs EΔ(BSA:PAL): $p = 0.0105$. Source data are available online for this figure.

 © The Author(s)

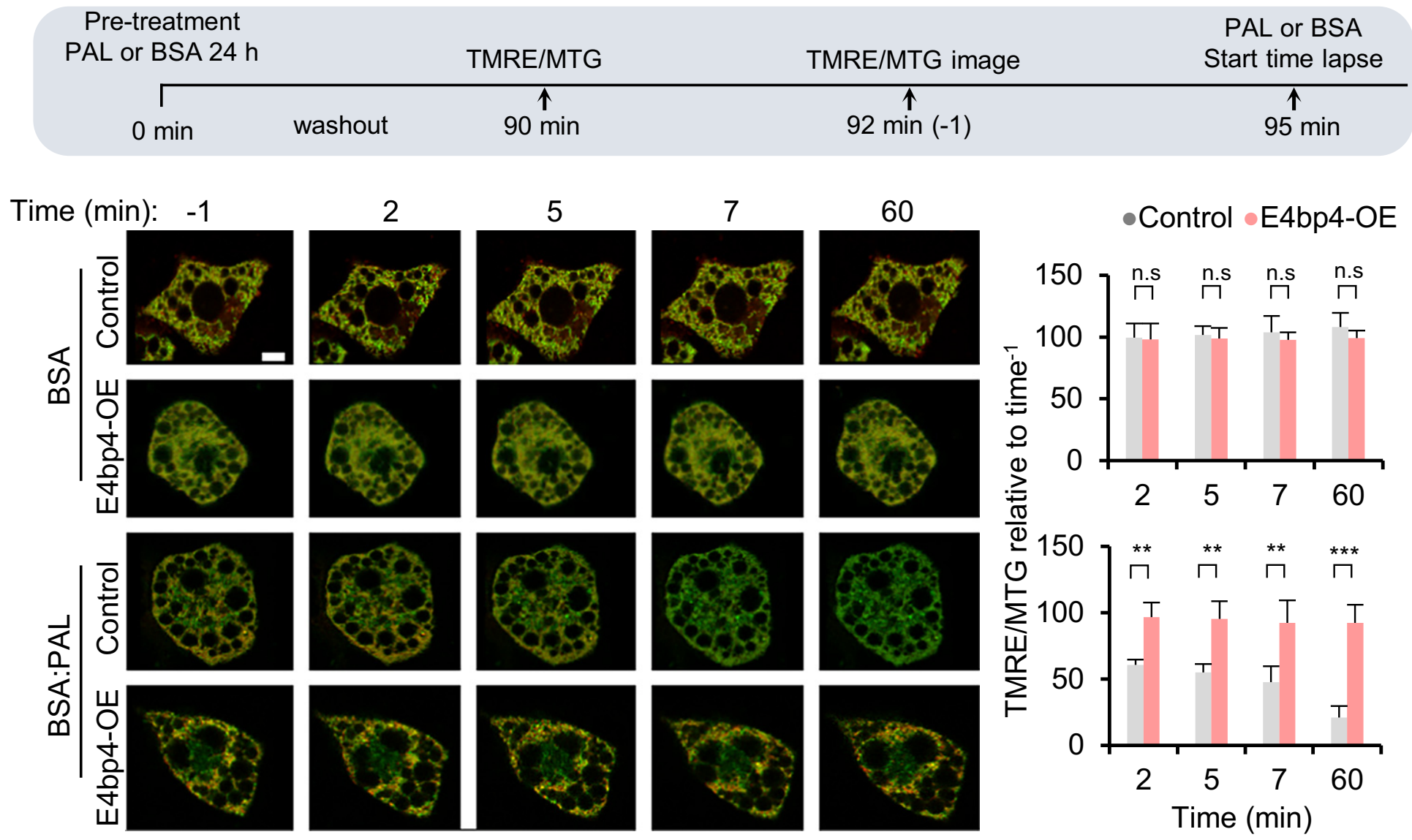

Figure EV5. Mitochondrial membrane potential in E4bp4-overexpressing brown adipocytes.

Top: Schematic illustration of the experimental design. Bottom left: Wave-like mitochondrial depolarization in primary brown adipocytes infected with AAV-Control and E4bp4 stained with TMRE (red) and MTG (green) stimulated with palmitate. Time-lapse imaging was performed at the onset of mitochondrial depolarization. Scale bar 5 µm. Bottom right: Quantification of depolarization occurring pre- and post-mitochondrial fragmentation (relative to minute -1). $n = 3$ per group. Data were presented as mean ± SEM. Statistical significance was determined by two-way ANOVA followed by Tukey's post hoc test. P values for each comparison within the BSA group follow: Control vs 2 min: $p = 0.245$; control vs 5 min: $p = 0.505$; control vs 7 min: $p = 0.364$; control vs 60 min: $p = 0.415$. P values for each comparison within BSA:PAL group follow: Control vs 2 min: $p = 0.003$; control vs 5 min: $p = 0.002$; control vs 7 min: $p = 0.004$; control vs 60 min: $p < 0.0001$. Source data are available online for this figure.

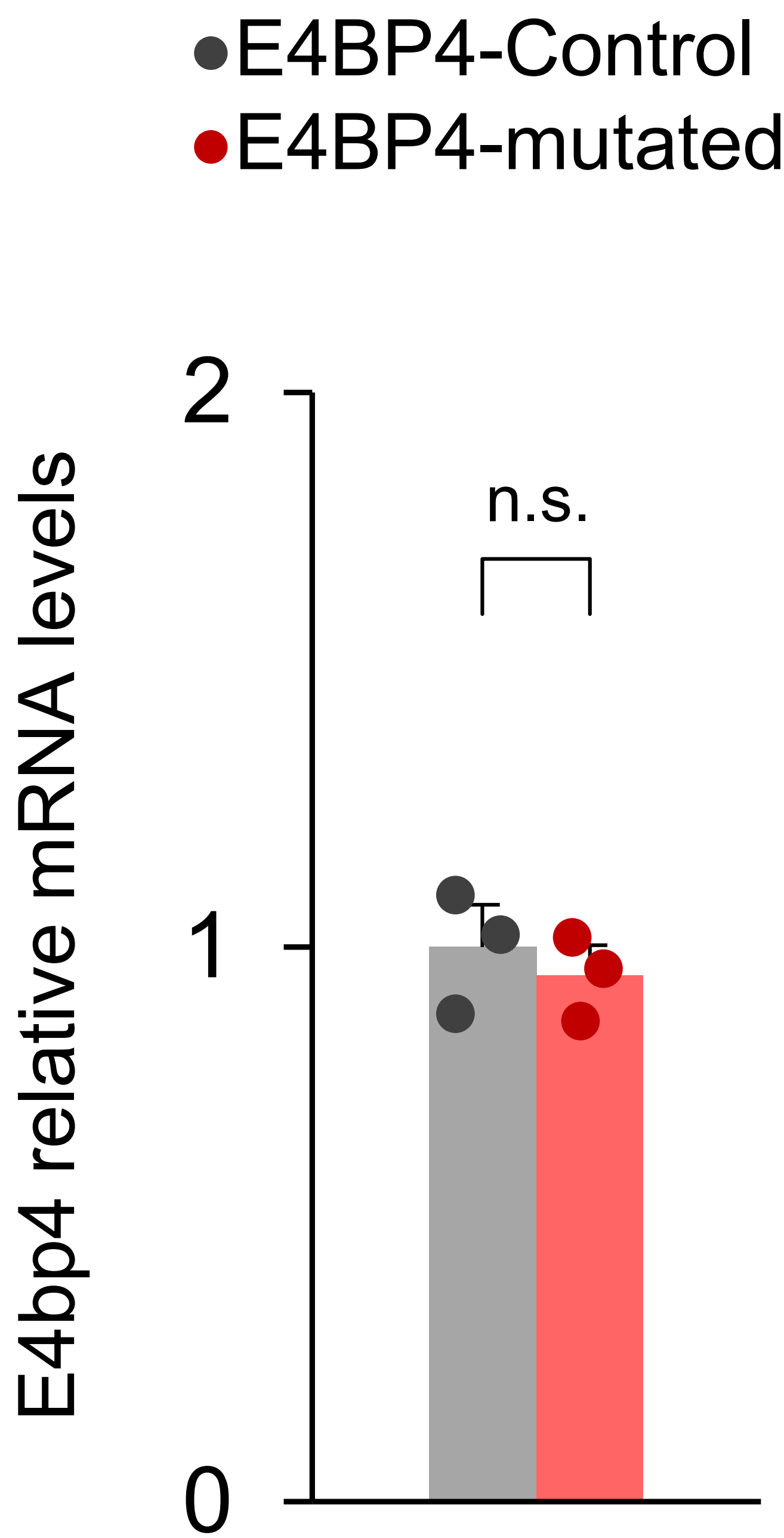

Figure EV6. Quantification of E4bp4 mRNA levels.

Relative mRNA levels of E4bp4 in brown adipocytes from E4BP4 and E4BP4-mutated cells. $n = 3$ per group. Data are presented as mean ± SEM. Two-sided P values were calculated using an unpaired Student's t-test. P value $= 0.454$. ns not significant. Source data are available online for this figure.

 © The Author(s)
