# Supplementary material for: E4BP4 safeguards brown fat mitochondria from obesity-induced fragmentation via ceramide repression
Source: EMBO Rep. 2026 Jun 22;27(15):4376–411. doi: 10.1038/s44319-026-00826-0 (PMC13458363; doi:10.1038/s44319-026-00826-0)
Supplement: Supplementary file 3 [file 44319_2026_826_MOESM2_ESM.pdf]

Table EV2. Primer sequences and probes for qRT-PCR.

Mouse qPCR primers

Gene	Forward primer	Reverse primer	Taqman probe
E4bp4	TTCCCCCTCACGGACCAGGGA	CATGGCGTCTTTCTTCTCGTCCGG	
Prdm16	CAGCACGGTGAAGCCATTC	GCGTGCATCCGCTTGTG	
Cers2	TCTTCTCAAAAAGTTCCGAG	AGTGATGATGAAAACGAATGG	
Cers3	CCTGGCTGCTATTAGTCTGATG	CTGCTTCCATCCAGCATAGG	
Cers4	TGTCGTTCAGCTTGAGTGAG	AGCAGGCTTCACAGAATTTC	
Cers5	CTCCAACGCTCACGAAATTTC	ATGCAGACAGAAGATGAGTG	
Sptlc1	TACTCAGAGACCTCCAGCTG	CACCAGGGATATGCTGTCATC	
Sptlc2	GGAGATGCTGAAGCGGAAC	GTATGAGCTGCTGACAGGCA	
Tbp	ACCCTTCACCAATGACTCCTATG	TGACTGCAGCAAATCGCTTGG	
36b4	TCCAGGCTTTGGGCATCA	CTTTATCAGCTGCACATCACTCAGA	
Ucp1	CACCTTCCCGCTGGACACT	CCCTAGGACACCTTTATACCTAATGG	
Tfam	ATTCCGAAGTGTTTTTCCAGCA	TCTGAAAGTTTTGCATCTGGGT	
Nrf1	AGCACGGAGTGACCCAAAC	TGTACGTGGCTACATGGACCT	
Cox8b	TGTGGGGATCTCAGCCATAGT	AGTGGGCTAAGACCCATCCTG	
Cers6	GTTCGGAGCATTCAACGCTG	CTGAGTCGTGAAGACAGAGG	Mm00556165_m1
Rplp0			Mm01974474_gH
Sg1-Enhancer	CACCGGATATGAAAGTCTTTCGAAA	AAACTTTCGAAAGACTTTCATATCC	
Sg2-enhancer	CACCGAAACTATTAAGTAATTCTGC	AAACGCAGAATTACTTAATAGTTTC	
Sg3-enhancer	CACCGCTGTGTGTCACAATTATGAG	AAACCTCATAATTGTGACACACAGC	
Sg4-enhancer	CACCAATTTCAACATTAGGAAAC	AAACGTTTCCTAATGTTGAAATT	
Sg5-enhancer	CACCGCCTTGTAAACATGTCCATT	AATGGACATGTTTACAAGGC	
qPCR-enhancer	GCCACGGTCGAGTCCATGAATG	CTGATTACCAAATACCTTGCC	
Sequencing-enhancer	GGGTACCCAGCAGTGCTAATACC	GGGCTACAGGCACTTTTGACAGC	
Sh-E4bp4	CCGGGCTTCGGACTCCAGGTAAATG CTCGAGCATTTACCTGGAGTCCGAAG CTTTTTG	AATTCAAAAAGCTTCGGACTCCAGGT AAATGCTCGAGCATTTACCTG GAGTCCGAAGC	
Mutagenesis primers	GGAAAAACAAGGGAGCTGCCAAAAG	GCCGTTTCTCCCAATACATG	

Human qPCR primers

Gene	Forward primer	Reverse primer
E4BP4	TCCTCAGTAGAACACACGCAG	TCATCTCTTGGCTCCCTTGT
TBP	CGGCTGTTTAACTTCGCTTCC	TGGGTTATCTTCACACGCCAAG
DIO2	GGAAGAGCTTCCTCCTCGAT	CTGGAGACATGCACCACACT
