# Supplementary material for: E4BP4 safeguards brown fat mitochondria from obesity-induced fragmentation via ceramide repression
Source: EMBO Rep. 2026 Jun 22;27(15):4376–411. doi: 10.1038/s44319-026-00826-0 (PMC13458363; doi:10.1038/s44319-026-00826-0)
Supplement: Supplementary file 4 [file 44319_2026_826_MOESM3_ESM.pdf]

EMBO reports

E4BP4 Safeguards Brown Fat Mitochondria from Obesity-Induced Fragmentation via Ceramide Repression

Carlos Sponton, Fernando Valdivieso-Rivera, Vanessa Furino, Carlos Eduardo Leher, Ariane Maria Zanesco, Monara Cruz, Flávia Gan, Adriana Santoro, Lara Regina-Ferreira, Giovanna Santos, Tiago Gonçalves, Luiz Leiria, Pedro Moraes-Vieira, Roger Castilho, Shingo Kajimura, Marcelo Mori, and Licio Velloso

Corresponding author(s): Carlos Sponton (csponton@usp.br)

Review Timeline:

Transfer Date:	1st Oct 25
Editorial Decision:	4th Nov 25
Revision Received:	6th Feb 26
Editorial Decision:	23rd Apr 26
Revision Received:	30th Apr 26
Accepted:	26th May 26

Editor: Kurt Weir

Transaction Report: This manuscript was transferred to EMBO Reports following peer review at Review Commons.

Review Commons

(Note: With the exception of the correction of typographical or spelling errors that could be a source of ambiguity, letters and reports are not edited. Depending on transfer agreements, referee reports obtained elsewhere may or may not be included in this compilation. Referee reports are anonymous unless the Referee chooses to sign their reports.)

Review #1

1. Evidence, reproducibility and clarity:

Evidence, reproducibility and clarity (Required)

Summary of the key results:

Valdivieso-Rivera and colleagues present a novel regulatory mechanism by which E4BP4 modulates C16:0 ceramide production in brown adipocytes. Several points warrant clarification or additional data.

Suggested improvements:

1) Figure 1F: There is an unexpected dip in gene expression at cold exposure days 3 and 7, followed by a rebound at day 14. Is this fluctuation biologically meaningful or technical?

2) Figures B: Sample size of EE experiments is too low to draw any meaningful conclusions or to know for certain if the data are reproducible. Small sample sizes, likely coming from one litter and one batch of AAV are prone to type I error.

3) Figures 2H and 2I (GTT): How was the AUC calculated? The GTT and ITT curves appear largely parallel aside from fasting differences. If total AUC was used instead of incremental AUC, it may overstate group differences. The recommended method is outlined in [DOI: 10.1038/s42255-021-00414-7]. Also, since insulin's half-life is ~10 minutes, later differences in the ITT curve likely reflect counterregulatory responses driven by hepatic gluconeogenesis.

4) Figure 3I: Why do cells (none of the groups) show no response to NE stimulation? Please clarify or provide potential mechanistic insight. Perhaps the cells were not differentiated well.

5) Figure 4F: How was mitochondrial fragmentation quantified? Please ensure that the ROI boxes shown in zoomed panels match the same region in size and shape - this applies throughout the manuscript.

5) Figures 3I vs 5N: There is a striking discrepancy between these panels. In both, cells were treated with palmitate for 6 h, yet the NE and CCCP responses differ significantly. Are these the same cell types and conditions? Please reconcile the differences.

6) Figure 3A: The claim that one group contains smaller mitochondria is not convincing. Both small and elongated mitochondria appear in each group. Moreover, it is unclear whether these minor differences are of any physiological relevance or whether they drive phenotypes.

7) Figure 3E: The claim that confocal microscopy reveals palmitate-induced mitochondrial fragmentation is difficult to discern. The images lack clear morphological differences.

8) Figure 3G: Dendra2-labeled mitochondria appear unaffected by palmitate, raising concern about the robustness of the effect across readouts.

9) Figure 5H: Were E4BP4 expression levels equivalent between WT and mutant cells? Quantification should be shown. Figure 5H: Were E4BP4 expression levels equivalent between WT and mutant cells? Quantification should be shown.

Cross-commenting

I agree with R2's points

2. Significance:

Significance (Required)

This advance is incremental for the basic science community.

3. How much time do you estimate the authors will need to complete the suggested revisions:

Estimated time to Complete Revisions (Required)

(Decision Recommendation)

Between 1 and 3 months

4. *Review Commons* values the work of reviewers and encourages them to get credit for their work. Select 'Yes' below to register your reviewing activity at [Web of Science Reviewer Recognition Service] (formerly Publons); note that the content of your review will not be visible on Web of Science.

Web of Science Reviewer Recognition

Yes

Review #2

1. Evidence, reproducibility and clarity:

Evidence, reproducibility and clarity (Required)

Summary: The manuscript by Valdivieso-Rivera et al. investigated the role of a transcription factor, E4BP4, in brown fat functions. Using in vivo AAV gain-of-function studies, in vitro primary cultured brown adipocytes, and transcription regulation studies, authors identified that E4BP4 works together with PRDM16 to suppress Cers6 transcriptions and its derived ceramide C16:0 production. The resulted decreasing C16:0 prevents diet-induced mitochondrial fragmentation within brown adipocytes, thereby promoting brown fat functions. Overall, this study employed state-of-the-art methodologies and the collected evidence generally supported the conclusion. However, there are issues remaining to be addressed.

Major Comments:

1. The evidence of mitochondrial fragmentation is not convincing. In the reviewer's opinion, Figures 3E, 3G, 4F, and 5M demonstrated a decrease in mitochondrial quantity, but not fragmentation.
2. Whether E4BP4-OE affects cold tolerance in mice is now shown.
3. A key experiment is missing: does adding C16:0 block the mitochondrial benefits of E4BP4-OE?
4. Whether PRDM16-OE mimics the effects of E4BP4 to induce p-Drp1 is not shown.

Minor points:

1. It is confusing whether the association shown in Figure 1C is a positive or an inverse association.
2. Results from the PRDM16-OE model were mostly obtained in cultured brown adipocytes. It would be worthwhile to investigate whether in vivo knockdown of E4BP4 blunts the Cers6-suppressing effects of PRDM16-OE.

Cross-commenting

Reviewer #1's comments are all solid, and I agree with all of them.

2. Significance:

Significance (Required)

Key strengths include state-of-the-art methodologies and detailed mechanistic studies. Key limitations include some unconvincing staining data, lack of key "rescue" experiments, and less novelty in molecular mechanisms (the ceramide-Drp1 pathway).

Overall, this study uncovers a critical role of E4BP4 in maintaining brown adipocyte mitochondrial integrity and function, advancing our understanding of TFs in brown fat biology. This study well fits readers' interests in the adipose biology and metabolism field.

3. How much time do you estimate the authors will need to complete the suggested revisions:

Estimated time to Complete Revisions (Required)

(Decision Recommendation)

Between 1 and 3 months

4. *Review Commons* values the work of reviewers and encourages them to get credit for their work. Select 'Yes' below to register your reviewing activity at Web of Science Reviewer Recognition Service (formerly Publons); note that the content of your review will not be visible on Web of Science.

Web of Science Reviewer Recognition

No

Revision Plan

Manuscript number: RC-2025-03113
Corresponding author(s): Carlos, Sponton

[The "revision plan" should delineate the revisions that authors intend to carry out in response to the points raised by the referees. It also provides the authors with the opportunity to explain their view of the paper and of the referee reports.

The document is important for the editors of affiliate journals when they make a first decision on the transferred manuscript. It will also be useful to readers of the reprint and help them to obtain a balanced view of the paper.

If you wish to submit a full revision, please use our "Full Revision" template. ***It is important to use the appropriate template to clearly inform the editors of your intentions.****]*

1. General Statements [optional]

This section is optional. Insert here any general statements you wish to make about the goal of the study or about the reviews.

We appreciate the reviewers' assessment of the significance of our work and would like to highlight where we believe the novelty of this study lies. Our findings identify E4BP4 as a key transcription factor that maintains mitochondrial homeostasis by restraining the overactivation of biological pathways – such as de novo ceramide synthesis – that are known to drive mitochondrial oxidative dysfunction in the context of obesity. We fully acknowledge that the link between C16:0 ceramide and mitochondrial fragmentation has been previously established. However, to our knowledge, our study is the first to connect this phenomenon to a transcriptional safeguard mechanism, thereby providing a new layer of understanding of how transcription factors preserve mitochondrial integrity and function in brown adipocytes. We believe this conceptual advance adds significant value to the field by framing E4BP4 as a transcriptional "guardian" of mitochondrial homeostasis.

2. Description of the planned revisions

Insert here a point-by-point reply that explains what revisions, additional experimentations and analyses are planned to address the points raised by the referees.

Reviewer #1 comment:
Figures B: Sample size of EE experiments is too low to draw any meaningful conclusions or to know for certain if the data are reproducible. Small sample sizes, likely coming from one litter and one batch of AAV are prone to type I error.

Response: We agree with reviewer observation that increasing sample size is essential to confirm reproducibility and robustness. We have therefore planned to repeat the EE experiments with a larger number of mice per group, derived from independent litters and AAV preparations, in order to strengthen the statistical power and validate the phenotype observed in the current study.

Reviewer #1 comment:
Figure 3I: Why do cells (none of the groups) show no response to NE stimulation? Please clarify or provide potential mechanistic insight. Perhaps the cells were not differentiated well.

Response: We agree that the absence of a robust NE response in Figure 3I requires further clarification. To address this, we have planned to repeat the in vitro oxygen consumption assay to confirm the phenotype presented in the study.

Reviewer #1 comment: Figures 3I vs 5N. There is a striking discrepancy between these panels. In both, cells were treated with palmitate for 6 h, yet the NE and CCCP responses differ significantly. Are these the same cell types and conditions? Please reconcile the differences.

Response: We would like to clarify that Figures 3I and 5N represent different experimental systems: Figure 3I shows data from primary brown adipocytes with E4bp4 transgene overexpression, whereas Figure 5N shows data from immortalized brown adipocytes with Cas9-mediated mutation of a 65 kb Cers6 enhancer site. Given the distinct cell types and genetic manipulations, a direct comparison between these two panels is not appropriate. Nevertheless, we agree that confirming the consistency of the phenotype across systems is important. To address this, we have planned to repeat oxygen consumption assays in both models to further validate the reproducibility of the observed effects.

Reviewer #2 comment: A key experiment is missing: does adding C16:0 block the mitochondrial benefits of E4BP4-OE?

Response: We thank the reviewer for this excellent suggestion. We agree that a rescue experiment is important to directly test whether C16:0 affects the mitochondrial benefits of E4BP4. To address this, we have planned to perform a co-overexpression of E4bp4 and Cers6 in brown adipocytes. The readouts will include mitochondrial morphology and oxygen consumption, enabling us to determine whether restoration of C16:0 production mitigates the protective mitochondria effects of E4BP4 overexpression. This experiment will provide direct mechanistic confirmation of the proposed model.

Reviewer #2 comment: Whether PRDM16-OE mimics the effects of E4BP4 to induce p-Drp1 is not shown.

Response: We thank the reviewer for this valuable suggestion. We agree that testing whether PRDM16 overexpression mimics the effects of E4BP4 on p-Drp1 is important to strengthen the mechanistic link between these transcription factors in terms of regulation of mitochondrial fragmentation. To address this, we have planned to include a Western blot analysis of p-Drp1 in the PRDM16-OE in brown adipocytes.

3. Description of the revisions that have already been incorporated in the transferred manuscript

Please insert a point-by-point reply describing the revisions that were already carried out and included in the transferred manuscript. If no revisions have been carried out yet, please leave this section empty.

Reviewer #1 comment:
Figure 1F: There is an unexpected dip in gene expression at cold exposure days 3 and 7, followed by a rebound at day 14. Is this fluctuation biologically meaningful or technical?

Response: We thank the reviewer for this thoughtful observation. A previous study demonstrated that E4bp4 (Nfil3) expression displays an early increase (at 2 hours), followed by a decrease in magnitude – while still remaining significantly higher than control – during beige adipocyte differentiation in response to forskolin treatment (DOI: 10.1016/j.molmet.2022.101619). The authors of that study suggested that E4bp4 may contribute to a second wave of cAMP-driven beige adipocyte differentiation. However, in the context of our work, further discussion on whether the fluctuations in BAT E4bp4 expression observed during cold exposure reflect biological regulation would be speculative. Importantly, despite these oscillations across time points, E4bp4 expression remained statistically significant compared with control, supporting the robustness of our findings. We have now introduced this observation in the Results section of the revised manuscript.

Reviewer #1 comment: Figures 2H and 2I (GTT): How was the AUC calculated? The GTT and ITT curves appear largely parallel aside from fasting differences. If total AUC was used instead of incremental AUC, it may overstate group differences. The recommended method is outlined in [DOI: 10.1038/s42255-021-00414-7]. Also, since insulin's half-life is ~10 minutes, later differences in the ITT curve likely reflect counterregulatory responses driven by hepatic gluconeogenesis.

Response: We would like to clarify that in our original manuscript we had already calculated the area of the curve (AOC) rather than the area under the curve (AUC), following the recommended approach (DOI: 10.1038/s42255-021-00414-7). Specifically, the AOC was derived by subtracting the baseline glucose value from each subsequent time point, ensuring that the analysis reflects

incremental changes rather than absolute glucose levels. We have now made this description more explicit in the revised version to avoid any ambiguity.

Reviewer #1 comment: Figure 4F: How was mitochondrial fragmentation quantified? Please ensure that the ROI boxes shown in zoomed panels match the same region in size and shape - this applies throughout the manuscript.

Response: We thank the reviewer for this valuable comment. To improve the quality and interpretation of the data, we have now included a quantitative analysis of mitochondrial morphology parameters associated with Figure 4F **(Figure S4B)**. Specifically, we analyzed:

- **Mitochondrial volume (μm³):** reflecting overall mitochondrial size.
- **Surface area (μm²):** reflecting membrane expansion.
- **Sphericity index:** indicating morphological rounding, which increases with fragmentation.
- **Number of branches and branch junctions per mitochondrion:** reflecting mitochondrial networking and fusion.

Related to Figure 4F

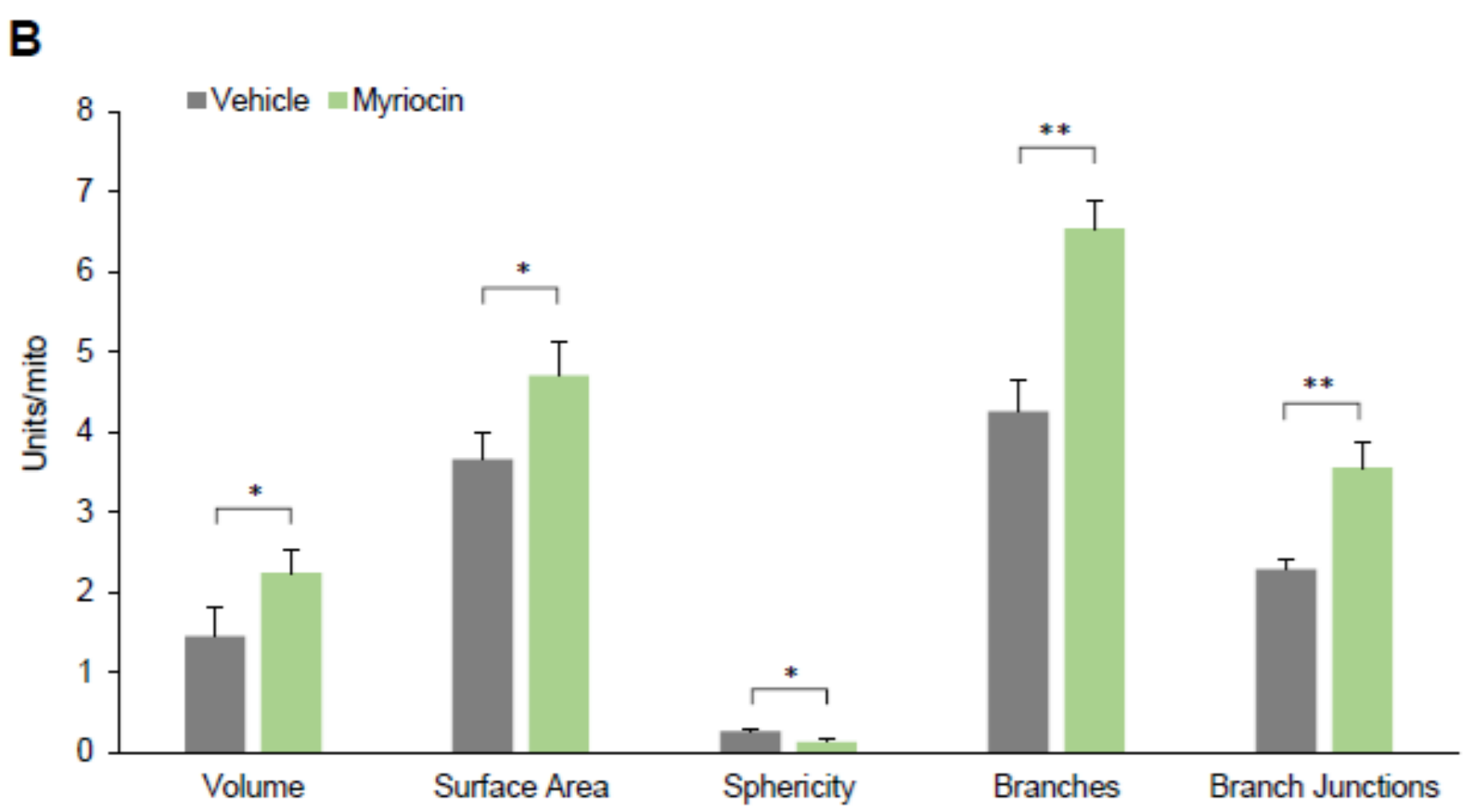

Myriocin treatment preserved mitochondrial volume and surface area, reduced sphericity, and increased both the number of branches and branch junctions, reflecting maintenance of a more interconnected mitochondrial network.

Additionally, we verified that the ROI boxes shown in the zoomed panels are consistent in both size and shape across groups, as requested. We have now introduced this observation in the Methods section of the revised manuscript.

Reviewer #1 comment: Figure 3A: The claim that one group contains smaller mitochondria is not convincing. Both small and elongated mitochondria appear in each group. Moreover, it is unclear whether these minor differences are of any physiological relevance or whether they drive phenotypes.

Response: We respectfully disagree with this observation and would like to clarify a few points.

1. We have already demonstrated a statistically significant difference in mitochondrial length between E4bp4-OE and control groups (Figures 3B and 3C). This was based on a random, unbiased analysis, which consistently confirmed longer mitochondria in E4bp4-OE compared with control.

2. Some degree of variability in mitochondrial length is expected in electron microscopy analyses, particularly because mitochondria from multiple cell types within iBAT are captured. It is important to note that the protective action of E4bp4 against mitochondrial fragmentation occurs specifically in brown adipocytes, where the transgene is expressed under the control of the adiponectin promoter.

3. To address the potential confounding heterogeneity of iBAT mitochondria, we performed complementary cell-autonomous analyses in vitro, allowing us to directly compare mitochondrial dynamics in E4bp4-OE versus control brown adipocytes. This analysis further confirmed that E4bp4-OE prevents lipid overload – induced mitochondrial fragmentation in brown adipocytes.

4. Finally, we emphasize that several studies have demonstrated that changes in mitochondrial dynamics, particularly under high-fat diet conditions, disrupt systemic energy homeostasis (DOI: 10.1016/j.cmet.2017.05.010; DOI: 10.1016/j.cell.2019.05.008; DOI: 10.1038/s42255-024-00978-0). Therefore, the differences we report are biologically meaningful in the broader context of mitochondrial dynamics and metabolic disease.

Reviewer #1 comment: Figure 3E: The claim that confocal microscopy reveals palmitate-induced mitochondrial fragmentation is difficult to discern. The images lack clear morphological differences.

Response: We thank the reviewer for this observation. To improve the interpretation of these results, we have now included a quantitative analysis of mitochondrial morphology parameters associated with **Figure 3E.** Specifically, we measured:

- **Mitochondrial volume (μm^3):** reflecting overall mitochondrial size.
- **Surface area (μm^2):** reflecting membrane expansion.
- **Sphericity index:** indicating morphological rounding, which increases with fragmentation.
- **Number of branches and branch junctions per mitochondrion:** reflecting mitochondrial networking and fusion.

As shown in the new analysis **(Figure S4A)**, palmitate treatment reduced mitochondrial volume, surface area, branches, and branch junctions, while increasing sphericity, consistent with a more fragmented phenotype in control cells. In contrast, these effects were significantly attenuated in E4bp4-OE cells, supporting our conclusion that E4BP4 overexpression protects against lipid

overload-induced mitochondrial fragmentation. This text was added in the Results section of the revised manuscript.

Related to Figure 3E

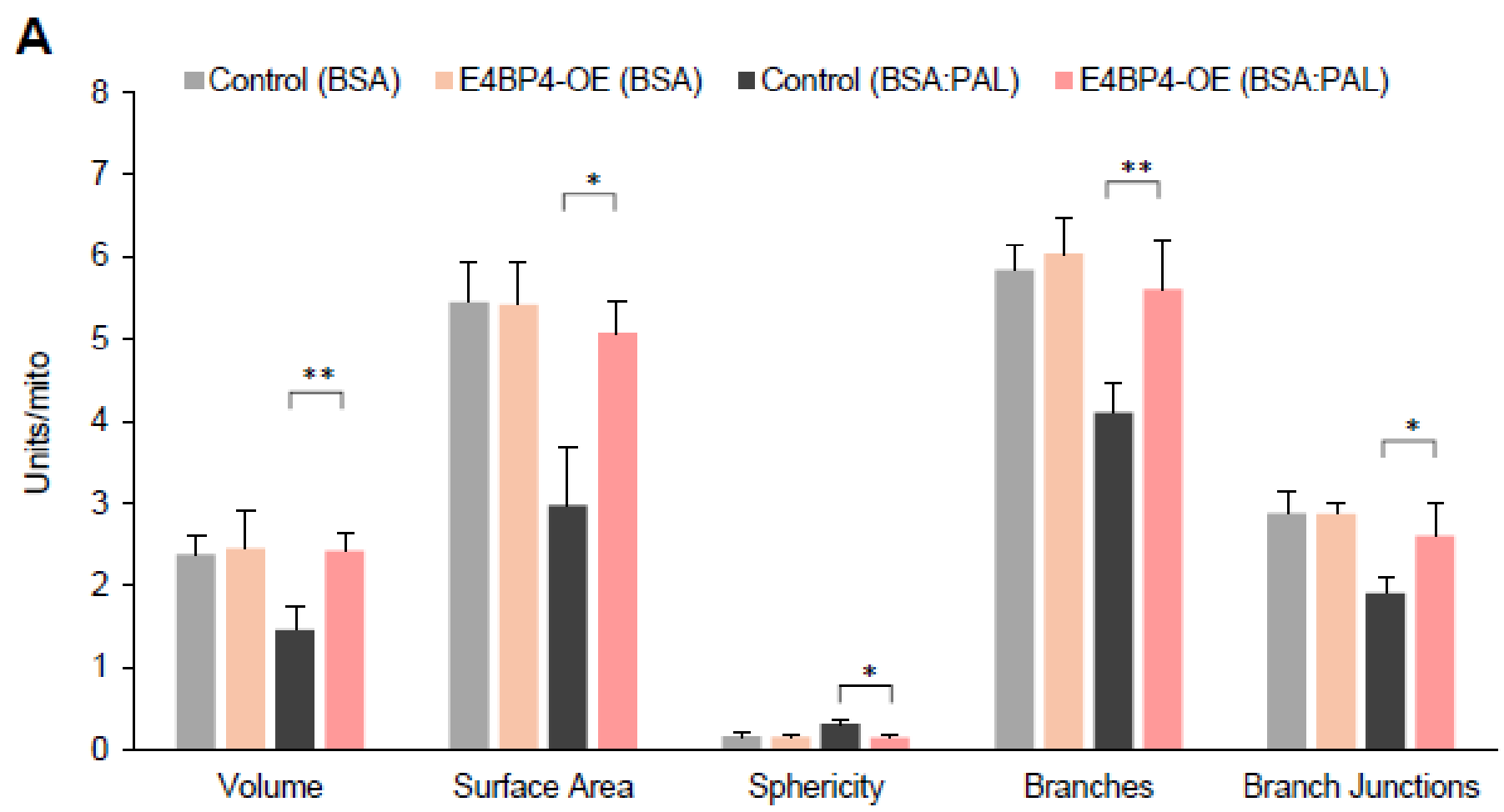

We believe this additional analysis strengthens the robustness of our findings and provides clear quantitative evidence for the morphological changes that were less apparent from qualitative image inspection alone.

Reviewer #1 comment: Figure 3G: Dendra2-labeled mitochondria appear unaffected by palmitate, raising concern about the robustness of the effect across readouts.

Response: We respectfully disagree with this observation. As shown in **Figure 3G** (bar graphs), palmitate-treated brown adipocytes exhibited a clear reduction in mitochondrial co-localization, which reflects lower levels of fused mitochondria, in the control group compared with E4bp4-OE. Importantly, no difference in mitochondrial co-localization was observed between the two groups under vehicle-treated conditions. This indicates that E4bp4 overexpression does not promote mitochondrial fusion per se, but rather prevents lipid overload – induced mitochondrial fragmentation. We also note that the representative images presented in **Figure 3G** are single snapshots taken from a time-lapse assay of mitochondrial dynamics. To further illustrate this effect, we direct the reviewer to the supplementary video accompanying this experiment, which clearly demonstrates the differences in mitochondrial behavior over time.

Reviewer #1 comment: Figure 5H: Were E4BP4 expression levels equivalent between WT and mutant cells? Quantification should be shown. Figure 5H: Were E4BP4 expression levels equivalent between WT and mutant cells? Quantification should be shown.

Response: We thank the reviewer for this important point. We have now added the quantification of E4bp4 mRNA levels in cells transduced with either the non-mutated vector (control) and the

vector carrying a mutation in the E4bp4 DNA-binding domain **(Figure S5)**. The data show no significant difference in E4bp4 expression between the two groups.

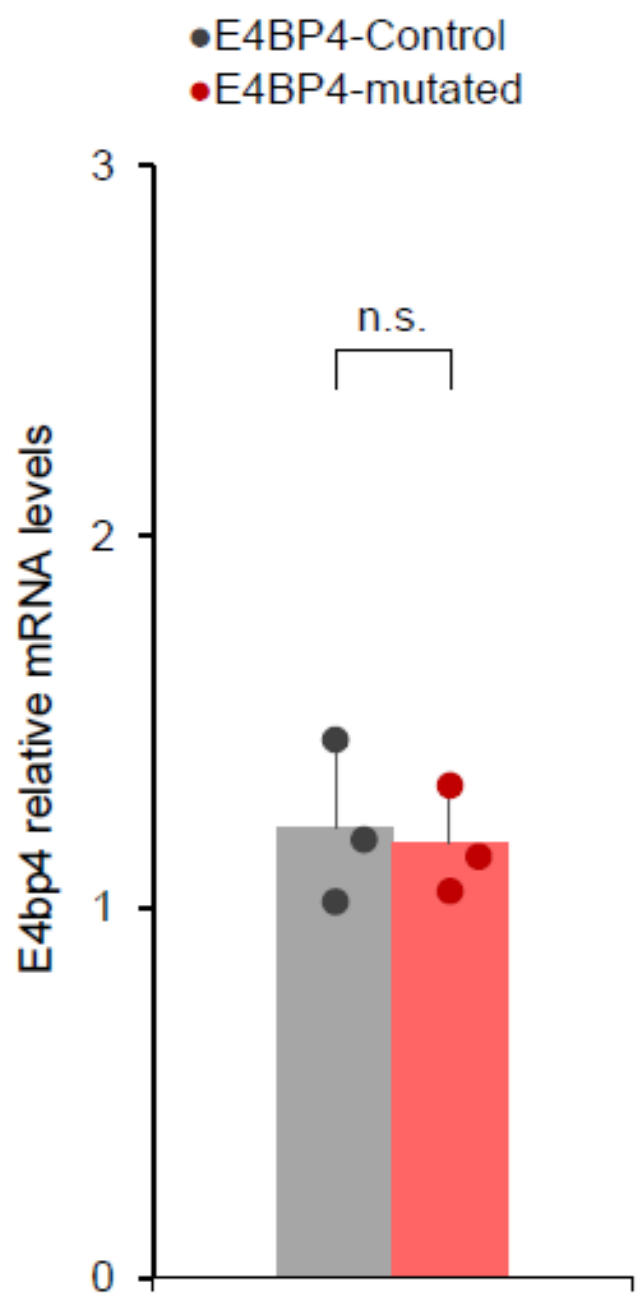

Reviewer #2 comment: The evidence of mitochondrial fragmentation is not convincing. In the reviewer's opinion, Figures 3E, 3G, 4F, and 5M demonstrated a decrease in mitochondrial quantity, but not fragmentation.

Response: We thank the reviewer for this observation. We have already addressed the comments from reviewer #1 (above) regarding Figures 3E, 3G and 4F related to measurements of mitochondria fragmentation. To strengthen the interpretation of these results, we have also performed a quantitative analysis of mitochondrial morphology parameters associated with **Figure 5M**. Specifically, we measured:

- **Mitochondrial volume (µm³):** reflecting overall mitochondrial size.
- **Surface area (µm²):** reflecting membrane expansion.
- **Sphericity index:** indicating morphological rounding, which increases with fragmentation.
- **Number of branches and branch junctions per mitochondrion:** reflecting mitochondrial networking and fusion.

As shown in the new analysis **(Figure S4C)**, palmitate treatment significantly reduced mitochondrial volume, surface area, and branching, while increasing sphericity, consistent with enhanced mitochondrial fragmentation in control cells. Notably, these changes were significantly blunted in the Cers6 enhancer edited cells (EΔ), supporting our conclusion that disruption of Cers6 protects against lipid overload–induced mitochondrial fragmentation**.** This text was added in the Results section of the revised manuscript.

Related to Figure 5M

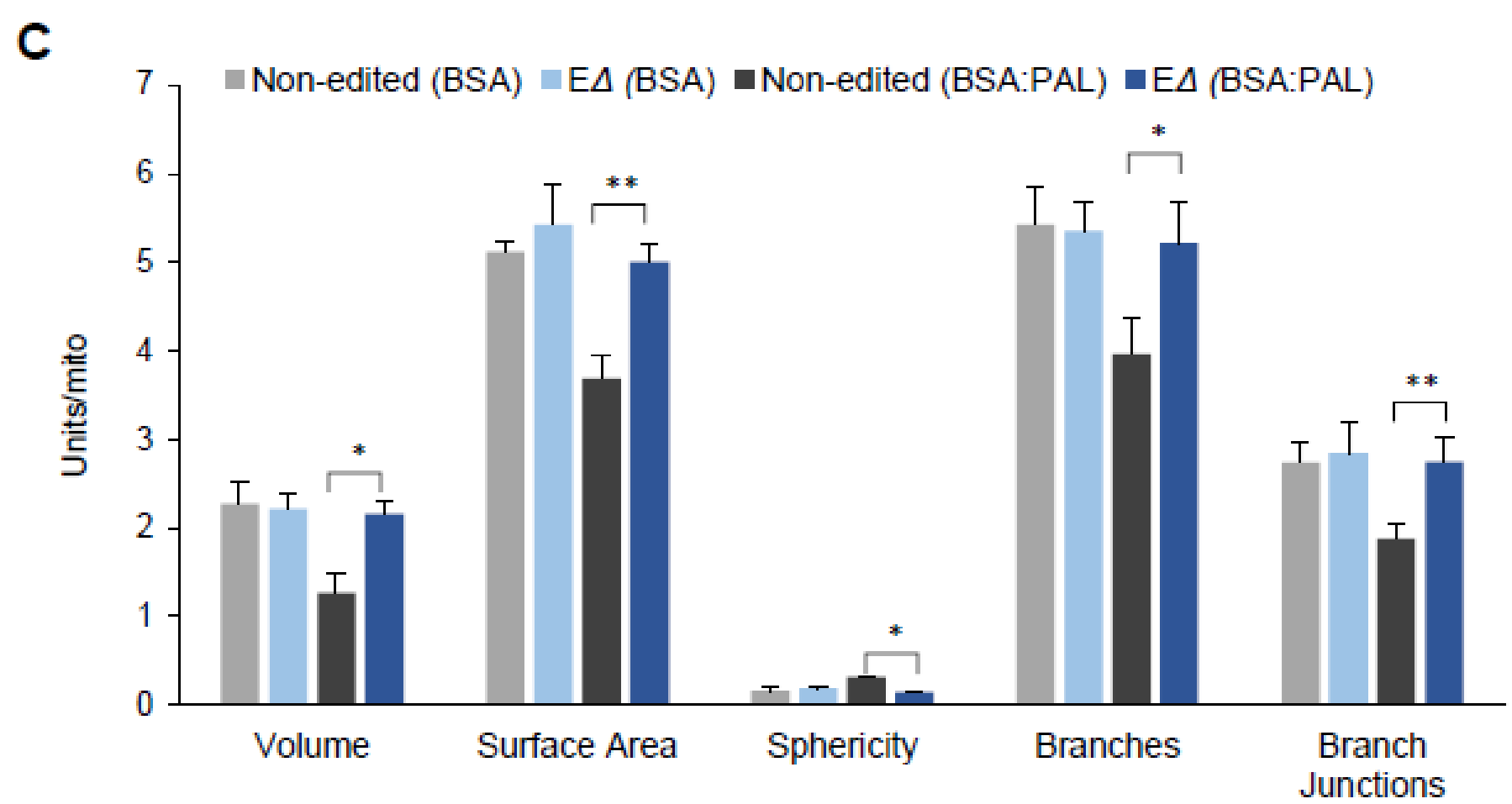

Regarding the reviewer's understanding of a "decrease in mitochondrial quantity, but not fragmentation," we respectfully disagree. The analyses performed for Figures 3E, 3G, 4F, and 5M clearly demonstrate that E4bp4 overexpression (E4bp4-OE) prevents lipid overload –induced mitochondrial fragmentation.

In relation to mitochondrial quantity, our data do not support differences in mitochondrial biogenesis between groups. Specifically, the expression of thermogenic and mitochondrial biogenesis genes **(Figure S2G)** as well as the mitochondrial-to-nuclear DNA ratio **(Figure S3D)** showed no significant changes, indicating that mitochondrial biogenesis is not altered.

Alternatively, it is possible that E4bp4 prevents mitophagy, as our results **(Figure 3H)** show that E4bp4-OE protects against lipid overload-induced mitochondrial depolarization. In this regard, previous studies have demonstrated that fragmented and depolarized mitochondria are targeted for degradation through mitophagy (DOI: 10.2337/db07-1781; DOI: 10.1074/jbc.M111.242412). While this explanation is consistent with our findings, we acknowledge that it remains speculative at this stage and, although interesting, is beyond the scope of the current study.

Reviewer #2 comment: It is confusing whether the association shown in Figure 1C is a positive or an inverse association.

Response: We thank the reviewer for pointing out this source of confusion. **Figure 1C** represents common variant associations for E4BP4, where the y-axis indicates the strength of association (-log10 p-value) rather than the direction (positive or inverse) of the effect. We have clarified this in the revised manuscript to avoid misinterpretation. The associations indicate that genetic variants in E4bp4 are positively linked with anthropometric traits such as weight, BMI, and waist–hip ratio.

Review COMMONS

5. Description of analyses that authors prefer not to carry out

Please include a point-by-point response explaining why some of the requested data or additional analyses might not be necessary or cannot be provided within the scope of a revision. This can be due to time or resource limitations or in case of disagreement about the necessity of such additional data given the scope of the study. Please leave empty if not applicable.

Reviewer #2 comment:
It would be worthwhile to investigate whether in vivo knockdown of E4BP4 blunts the Cers6-suppressing effects of PRDM16-OE.

Response: We agree that assessing in vivo loss-of-function of E4bp4 in the context of Prdm16 overexpression would be highly informative. At present, this experiment is technically not feasible, as it would require the generation and characterization of complex *in vivo* models beyond the scope of the current study. Nevertheless, we are actively considering this as a future direction. In the meantime, we believe that the *in vitro* experiments in brown adipocytes provided here are sufficient to establish the mechanistic relationship between E4BP4 and PRDM16 in the regulation of Cers6 expression.

Reviewer #2 comment: Whether E4BP4-OE affects cold tolerance in mice is now shown.

Response: We thank the reviewer for this thoughtful comment. In our study, we performed an iBAT-specific E4bp4 gain-of-function assay because we observed a downregulation of E4bp4 expression in the context of obesity. The rationale for this approach was to rescue E4bp4 expression in iBAT and thereby evaluate its systemic and mechanistic effects under obesogenic conditions. We recognize that a gain-of-function assay during cold challenge would further enhance E4bp4 expression and, while interesting, this would more directly address the role of E4bp4 in thermogenic regulation rather than in obesity-related metabolic dysfunction. For this reason, we believe that a detailed investigation of E4bp4 in cold-induced thermogenesis is an important but separate question that lies beyond the scope of the current study.

Dear Prof. Sponton

Thank you for the transfer of your research manuscript to our journal. I have now read and discussed your work with my colleagues here and assessed the accompanying referee reports from Review Commons. In addition, I have also consulted an expert editorial advisor, who is familiar both with the field and with our journal and its scope.

The Review Commons referees, as well as our scientific advisor, express interest in the work's value for the metabolism and adipose biology field.

Given this support and given that you plan to address all referee concerns and to further strengthen your data, we would like to invite you to revise your manuscript in line with your submitted revision plan. Please address all referee concerns in a complete point-by-point response. Acceptance of the manuscript will depend on a positive outcome of a second round of review. It is EMBO Reports policy to allow a single round of revision only and acceptance or rejection of the manuscript will therefore depend on the completeness of your responses included in the next, final version of the manuscript.

We realize that it is difficult to revise to a specific deadline. In the interest of protecting the conceptual advance provided by the work, we recommend a revision within 3 months (February 1st). Please discuss the revision progress ahead of this time with the editor if you require more time to complete the revisions.

I am also happy to discuss the revision further via e-mail or a video call, if you wish.

You can either publish the study as a short report or as a full article. For short reports, the revised manuscript should not exceed 27,000 characters (including spaces but excluding materials & methods and references) and 5 main plus 5 expanded view figures. The results and discussion sections must further be combined, which will help to shorten the manuscript text by eliminating some redundancy that is inevitable when discussing the same experiments twice. For a normal article there are no length limitations, but it should have more than 5 main figures and the results and discussion sections must be separate. In both cases, the entire materials and methods must be included in the main manuscript file.

================
IMPORTANT NOTE:

We perform an initial quality control of all revised manuscripts before re-review. Your manuscript will FAIL this control and the handling will be delayed IN CASE the following APPLIES:

1) A data availability section providing access to data deposited in public databases is missing. If you have not deposited any data, please add a sentence to the data availability section that explains that.

2) Your manuscript contains statistics and error bars based on n=2. Please use scatter blots in these cases. No statistics should be calculated if n=2.

When submitting your revised manuscript, please carefully review the instructions that follow below. Failure to include requested items will delay the evaluation of your revision.

================

When submitting your revised manuscript, we will require:

1) a .docx formatted version of the manuscript text (including legends for main figures, EV figures and tables). Please make sure that the changes are highlighted to be clearly visible.

2) individual production quality figure files as .eps, .tif, .jpg (one file per figure).
Please download our Figure Preparation Guidelines (figure preparation pdf) from our Author Guidelines pages https://www.embopress.org/page/journal/14693178/authorguide for more info on how to prepare your figures.

3) a .docx formatted letter INCLUDING the reviewers' reports and your detailed point-by-point responses to their comments. As part of the EMBO Press transparent editorial process, the point-by-point response is part of the Review Process File (RPF), which will be published alongside your paper.

4) a complete author checklist, which you can download from our author guidelines (). Please insert information in the checklist that is also reflected in the manuscript. The completed author checklist will also be part of the RPF.

5) Please note that all corresponding authors are required to supply an ORCID ID for their name upon submission of a revised manuscript (). Please find instructions on how to link your ORCID ID to your account in our manuscript tracking system in our Author guidelines
()

6) We replaced Supplementary Information with Expanded View (EV) Figures and Tables that are collapsible/expandable online. A maximum of 5 EV Figures can be typeset. EV Figures should be cited as 'Figure EV1, Figure EV2" etc... in the text and their respective legends should be included in the main text after the legends of regular figures.

- For the figures that you do NOT wish to display as Expanded View figures, they should be bundled together with their legends in a single PDF file called *Appendix*, which should start with a short Table of Content. Appendix figures should be referred to in the main text as: "Appendix Figure S1, Appendix Figure S2" etc. See detailed instructions regarding expanded view here:

- Additional Tables/Datasets should be labeled and referred to as Table EV1, Dataset EV1, etc. Legends have to be provided in a separate tab in case of .xls files. Alternatively, the legend can be supplied as a separate text file (README) and zipped together with the Table/Dataset file.

7) Please note that a Data Availability section at the end of Materials and Methods is now mandatory. In case you have no data that requires deposition in a public database, please state so instead of refereeing to the database.
See also < https://www.embopress.org/page/journal/14693178/authorguide#dataavailability>). Please note that the Data Availability Section is restricted to new primary data that are part of this study.

8) At EMBO Press we ask authors to provide source data for the main manuscript figures. You will receive a separate email with instructions for providing source data with your revised manuscript, including how to upload and organize the files.

Additional information on source data and instruction on how to label the files are available

9) The journal requires a statement specifying whether or not authors have competing interests (defined as all potential or actual interests that could be perceived to influence the presentation or interpretation of an article). In case of competing interests, this must be specified in your disclosure statement. Further information: https://www.embopress.org/competing-interests

10) Figure legends and data quantification:
The following points must be specified in each figure legend:

- the name of the statistical test used to generate error bars and P values,
- the EXACT p-values,
- the number (n) of independent experiments (please specify technical or biological replicates) underlying each data point,
- the nature of the bars and error bars (s.d., s.e.m.)

- If the data are obtained from n {less than or equal to} 5, show the individual data points in addition to the SD or SEM.
- If the data are obtained from n {less than or equal to} 2, use scatter blots showing the individual data points.

Discussion of statistical methodology can be reported in the materials and methods section, but figure legends should contain a basic description of n, P and the test applied.

See also the guidelines for figure legend preparation:
https://www.embopress.org/page/journal/14693178/authorguide#figureformat

- Please also include scale bars in all microscopy images and define their size in the legend, not the image.

11) Our journal encourages inclusion of *data citations in the reference list* to directly cite datasets that were re-used and obtained from public databases. Data citations in the article text are distinct from normal bibliographical citations and should directly link to the database records from which the data can be accessed. In the main text, data citations are formatted as follows: "Data ref: Smith et al, 2001" or "Data ref: NCBI Sequence Read Archive PRJNA342805, 2017". In the Reference list, data citations must be labeled with "[DATASET]". A data reference must provide the database name, accession number/identifiers and a resolvable link to the landing page from which the data can be accessed at the end of the reference. Further instructions are available at .

12) All Materials and Methods need to be described in the main text using our 'Structured Methods' format. According to this format, the Methods section includes a Reagents and Tools Table (listing key reagents, experimental models, software and relevant equipment and including their sources and relevant identifiers) followed by a Methods and Protocols section describing the methods, ideally using a step-by-step protocol format. The aim is to facilitate adoption of the methodologies across labs. Please download and fill our Reagents and Tools Table template (.docx), which you can find in our author guidelines:

https://www.embopress.org/page/journal/14693178/authorguide#structuredmethods.
When submitting your revised manuscript, please do not include the Reagents and Tools Table in the Methods section of the manuscript but upload it as a separate file choosing the file type "Reagent Table".
An example of a Method paper with Structured Methods can be found here:
https://www.embopress.org/doi/10.15252/msb.20178071.

13) As part of the EMBO publication's Transparent Editorial Process, EMBO Reports publishes online a Review Process File to accompany accepted manuscripts. This File will be published in conjunction with your paper and will include the referee reports, your point-by-point response and all pertinent correspondence relating to the manuscript.

You are able to opt out of this by letting the editorial office know (emboreports@embo.org). If you do opt out, the Review Process File link will point to the following statement: "No Review Process File is available with this article, as the authors have chosen not to make the review process public in this case."

We would also welcome the submission of cover suggestions, or motifs to be used by our Graphics Illustrator in designing a cover.

I look forward to seeing a revised form of your manuscript when it is ready and please let me know if you have questions or comments regarding the revision.

Yours sincerely,

Kurt Weir
Editor
EMBO Reports

Manuscript number: RC-2025-03113
Corresponding author(s): Carlos, Sponton

[The "revision plan" should delineate the revisions that authors intend to carry out in response to the points raised by the referees. It also provides the authors with the opportunity to explain their view of the paper and of the referee reports.

The document is important for the editors of affiliate journals when they make a first decision on the transferred manuscript. It will also be useful to readers of the reprint and help them to obtain a balanced view of the paper.

If you wish to submit a full revision, please use our "Full Revision" template. ***It is important to use the appropriate template to clearly inform the editors of your intentions.****]*

1. General Statements [optional]

This section is optional. Insert here any general statements you wish to make about the goal of the study or about the reviews.

We appreciate the reviewers' assessment of the significance of our work and would like to highlight where we believe the novelty of this study lies. Our findings identify E4BP4 as a key transcription factor that maintains mitochondrial homeostasis by restraining the overactivation of biological pathways – such as de novo ceramide synthesis – that are known to drive mitochondrial oxidative dysfunction in the context of obesity. We fully acknowledge that the link between C16:0 ceramide and mitochondrial fragmentation has been previously established. However, to our knowledge, our study is the first to connect this phenomenon to a transcriptional safeguard mechanism, thereby providing a new layer of understanding of how transcription factors preserve mitochondrial integrity and function in brown adipocytes. We believe this conceptual advance adds significant value to the field by framing E4BP4 as a transcriptional "guardian" of mitochondrial homeostasis.

2. Description of the planned revisions

Insert here a point-by-point reply that explains what revisions, additional experimentations and analyses are planned to address the points raised by the referees.

All experiments proposed in response to the referees' comments have been fully performed and are described in detail in Section 3 of this letter. For clarity, we refer to experiments added after the first revision as part of the "final revision".

3. Description of the revisions that have already been incorporated in the transferred manuscript

Please insert a point-by-point reply describing the revisions that were already carried out and included in the transferred manuscript. If no revisions have been carried out yet, please leave this section empty.

Reviewer #1 comment:
Figure 1F: There is an unexpected dip in gene expression at cold exposure days 3 and 7, followed by a rebound at day 14. Is this fluctuation biologically meaningful or technical?

Response: We thank the reviewer for this thoughtful observation. A previous study demonstrated that E4bp4 (Nfil3) expression displays an early increase (at 2 hours), followed by a decrease in magnitude – while still remaining significantly higher than control – during beige adipocyte differentiation in response to forskolin treatment (DOI: 10.1016/j.molmet.2022.101619). The authors of that study suggested that E4bp4 may contribute to a second wave of cAMP-driven beige adipocyte differentiation. However, in the context of our work, further discussion on whether the fluctuations in BAT E4bp4 expression observed during cold exposure reflect biological regulation would be speculative. Importantly, despite these oscillations across time points, E4bp4 expression remained statistically significant compared with control, supporting the robustness of our findings. We have now introduced this observation in the Results section of the revised manuscript (lines 180-186).

Reviewer #1 comment: Figures 2H and 2I (GTT): How was the AUC calculated? The GTT and ITT curves appear largely parallel aside from fasting differences. If total AUC was used instead of incremental AUC, it may overstate group differences. The recommended method is outlined in [DOI: 10.1038/s42255-021-00414-7]. Also, since insulin's half-life is ~10 minutes, later differences in the ITT curve likely reflect counterregulatory responses driven by hepatic gluconeogenesis.

Response: We would like to clarify that in our original manuscript we had already calculated the area of the curve (AOC) rather than the area under the curve (AUC), following the recommended approach (DOI: 10.1038/s42255-021-00414-7). Specifically, the AOC was derived by subtracting the baseline glucose value from each subsequent time point, ensuring that the analysis reflects incremental changes rather than absolute glucose levels. We have now made this description more explicit in the revised version to avoid any ambiguity (lines 247-249).

Reviewer #1 comment: Figure 4F: How was mitochondrial fragmentation quantified? Please ensure that the ROI boxes shown in zoomed panels match the same region in size and shape - this applies throughout the manuscript.

Response: We thank the reviewer for this valuable comment. To improve the quality and interpretation of the data, we have now included a quantitative analysis of mitochondrial morphology parameters associated with Figure 4F **(Figure EV4B)**. Specifically, we analyzed:

•	**Mitochondrial volume (µm³):** reflecting overall mitochondrial size.
•	**Surface area (µm²):** reflecting membrane expansion.
•	**Sphericity index:** indicating morphological rounding, which increases with fragmentation.
•	**Number of branches and branch junctions per mitochondrion:** reflecting mitochondrial networking and fusion.

Related to Figure 4F

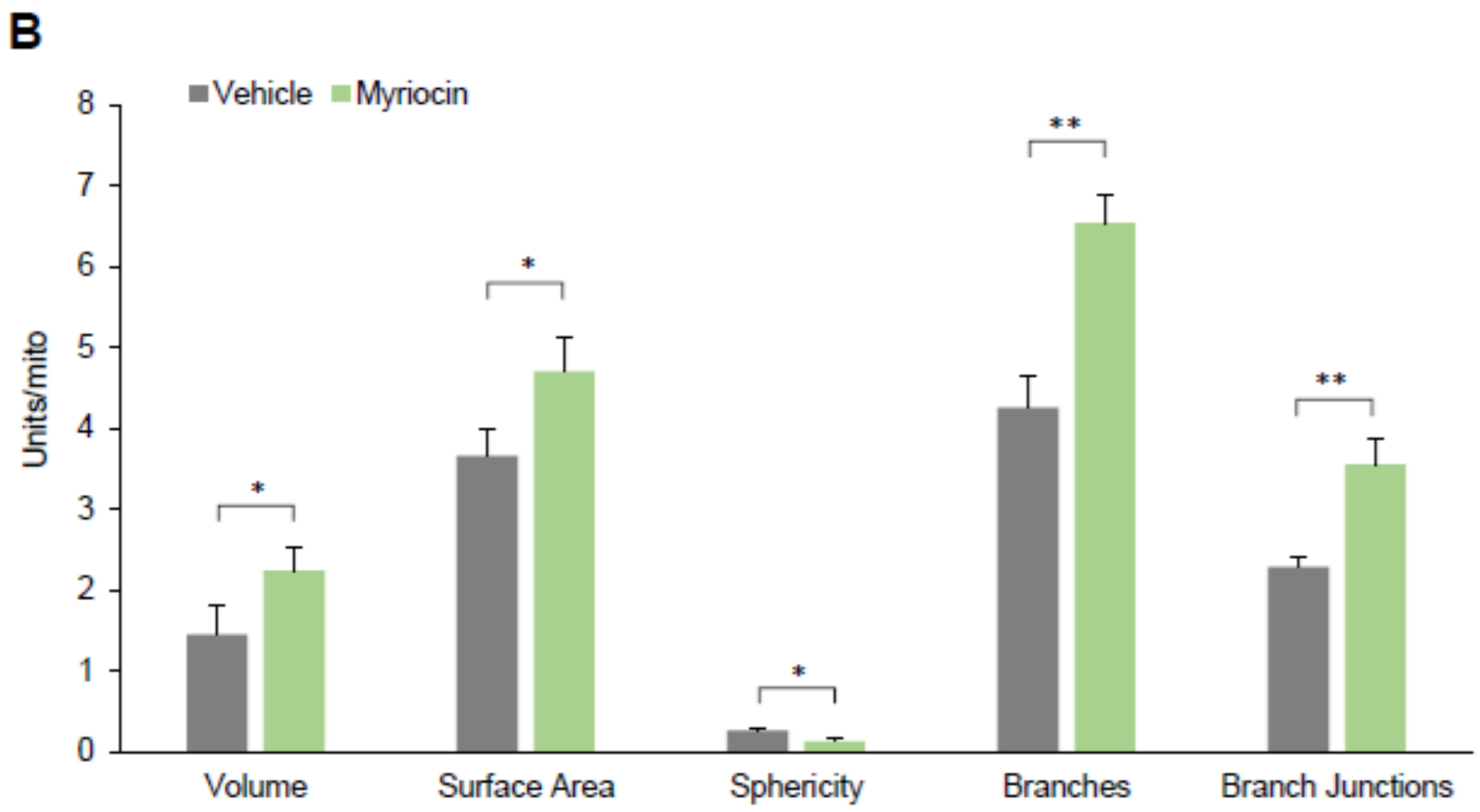

The new results are presented in **Figure EV4B**

Myriocin treatment preserved mitochondrial volume and surface area, reduced sphericity, and increased both the number of branches and branch junctions, reflecting maintenance of a more interconnected mitochondrial network.
Additionally, we verified that the ROI boxes shown in the zoomed panels are consistent in both size and shape across groups, as requested. We have now introduced this observation in manuscript (lines 375-378) and the Methods section of the revised manuscript.

Reviewer #1 comment: Figure 3A: The claim that one group contains smaller mitochondria is not convincing. Both small and elongated mitochondria appear in each group. Moreover, it is unclear whether these minor differences are of any physiological relevance or whether they drive phenotypes.

Response: We respectfully disagree with this observation and would like to clarify a few points:

1.	We have already demonstrated a statistically significant difference in mitochondrial length between E4bp4-OE and control groups **(Figures 3B and 3C)**. This was based on a random, unbiased analysis, which consistently confirmed longer mitochondria in E4bp4-OE compared with control.

2. Some degree of variability in mitochondrial length is expected in electron microscopy analyses, particularly because mitochondria from multiple cell types within iBAT are captured. It is important to note that the protective action of E4bp4 against mitochondrial fragmentation occurs specifically in brown adipocytes, where the transgene is expressed under the control of the adiponectin promoter.

3. To address the potential confounding heterogeneity of iBAT mitochondria, we performed complementary cell-autonomous analyses in vitro, allowing us to directly compare mitochondrial dynamics in E4bp4-OE versus control brown adipocytes. This analysis further confirmed that E4bp4-OE prevents lipid overload – induced mitochondrial fragmentation in brown adipocytes.

4. Finally, we emphasize that several studies have demonstrated that changes in mitochondrial dynamics, particularly under high-fat diet conditions, disrupt systemic energy homeostasis (DOI: 10.1016/j.cmet.2017.05.010; DOI: 10.1016/j.cell.2019.05.008; DOI: 10.1038/s42255-024-00978-0). Therefore, the differences we report are biologically meaningful in the broader context of mitochondrial dynamics and metabolic disease.

Reviewer #1 comment: Figure 3E: The claim that confocal microscopy reveals palmitate-induced mitochondrial fragmentation is difficult to discern. The images lack clear morphological differences.

Response: We thank the reviewer for this observation. To improve the interpretation of these results, we have now included a quantitative analysis of mitochondrial morphology parameters associated with **Figure 3E.** Specifically, we measured:

- **Mitochondrial volume (μm³):** reflecting overall mitochondrial size.
- **Surface area (μm²):** reflecting membrane expansion.
- **Sphericity index:** indicating morphological rounding, which increases with fragmentation.
- **Number of branches and branch junctions per mitochondrion:** reflecting mitochondrial networking and fusion.

As shown in the new analysis **(Figure EV4A)**, palmitate treatment reduced mitochondrial volume, surface area, branches, and branch junctions, while increasing sphericity, consistent with a more fragmented phenotype in control cells. In contrast, these effects were significantly attenuated in E4bp4-OE cells, supporting our conclusion that E4BP4 overexpression protects against lipid overload-induced mitochondrial fragmentation. This text was added in the Results section of the revised manuscript (lines 291-294).

Related to Figure 3E

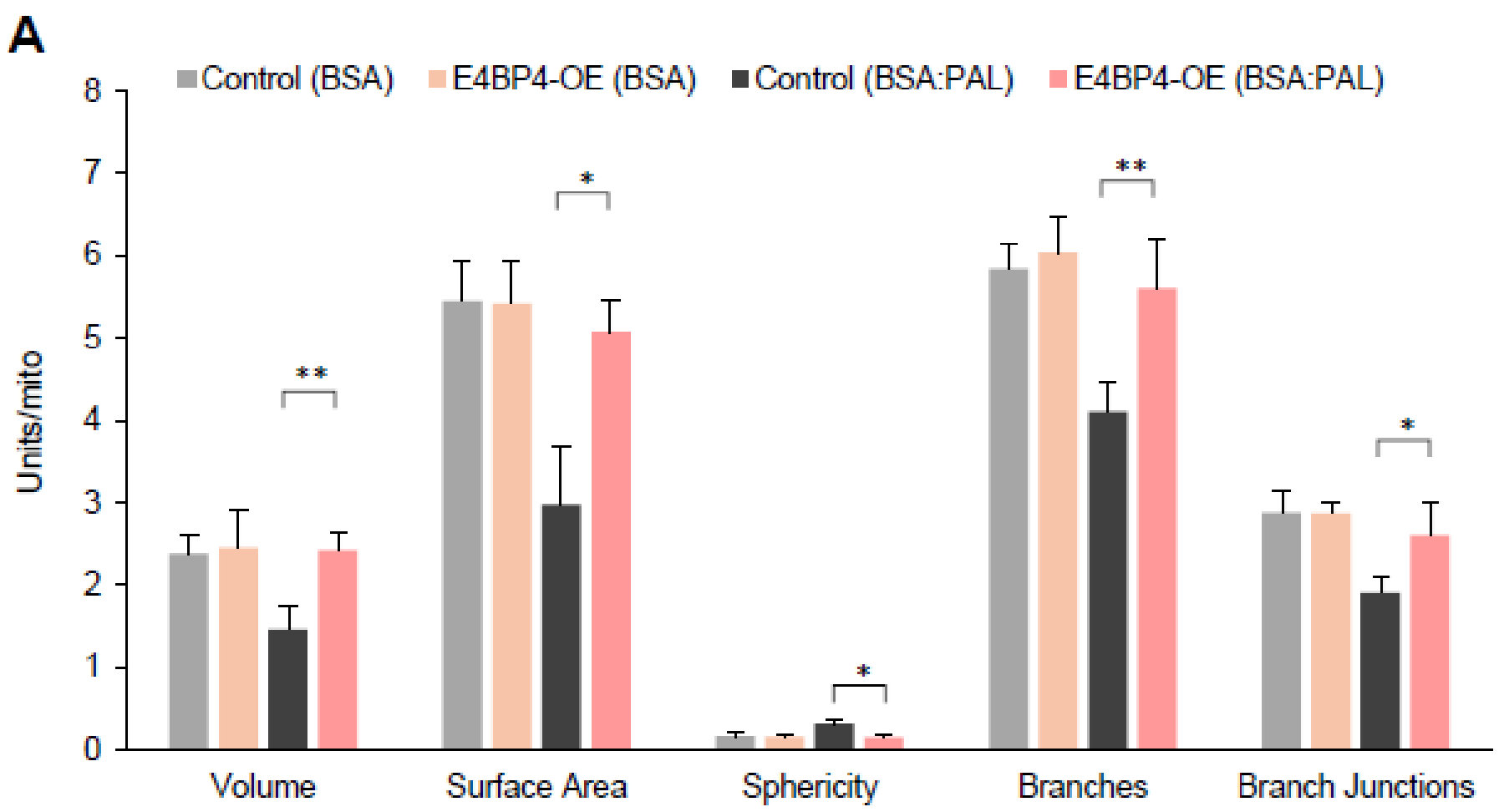

The new results are presented in **Figure EV4A**

We believe this additional analysis strengthens the robustness of our findings and provides clear quantitative evidence for the morphological changes that were less apparent from qualitative image inspection alone.

Reviewer #1 comment: Figure 3G: Dendra2-labeled mitochondria appear unaffected by palmitate, raising concern about the robustness of the effect across readouts.

Response: We respectfully disagree with this observation. As shown in **Figure 3G** (bar graphs), palmitate-treated brown adipocytes exhibited a clear reduction in mitochondrial co-localization, which reflects lower levels of fused mitochondria, in the control group compared with E4bp4-OE. Importantly, no difference in mitochondrial co-localization was observed between the two groups under vehicle-treated conditions. This indicates that E4bp4 overexpression does not promote mitochondrial fusion per se, but rather prevents lipid overload – induced mitochondrial fragmentation. We also note that the representative images presented in **Figure 3G** are single snapshots taken from a time-lapse assay of mitochondrial dynamics. To further illustrate this effect, we direct the reviewer to the supplementary video accompanying this experiment, which clearly demonstrates the differences in mitochondrial behavior over time.

Reviewer #1 comment: Figure 5H: Were E4BP4 expression levels equivalent between WT and mutant cells? Quantification should be shown. Figure 5H: Were E4BP4 expression levels equivalent between WT and mutant cells? Quantification should be shown.

Response: We thank the reviewer for this important point. We have now added the quantification of E4bp4 mRNA levels in cells transduced with either the non-mutated vector (control) and the vector carrying a mutation in the E4bp4 DNA-binding domain **(Appendix Fig. S1)**. The data show

no significant difference in E4bp4 expression between the two groups. Please note that in the revised manuscript (lines 444-446), **Figure 5H** has been renumbered as **Figure 6A**.

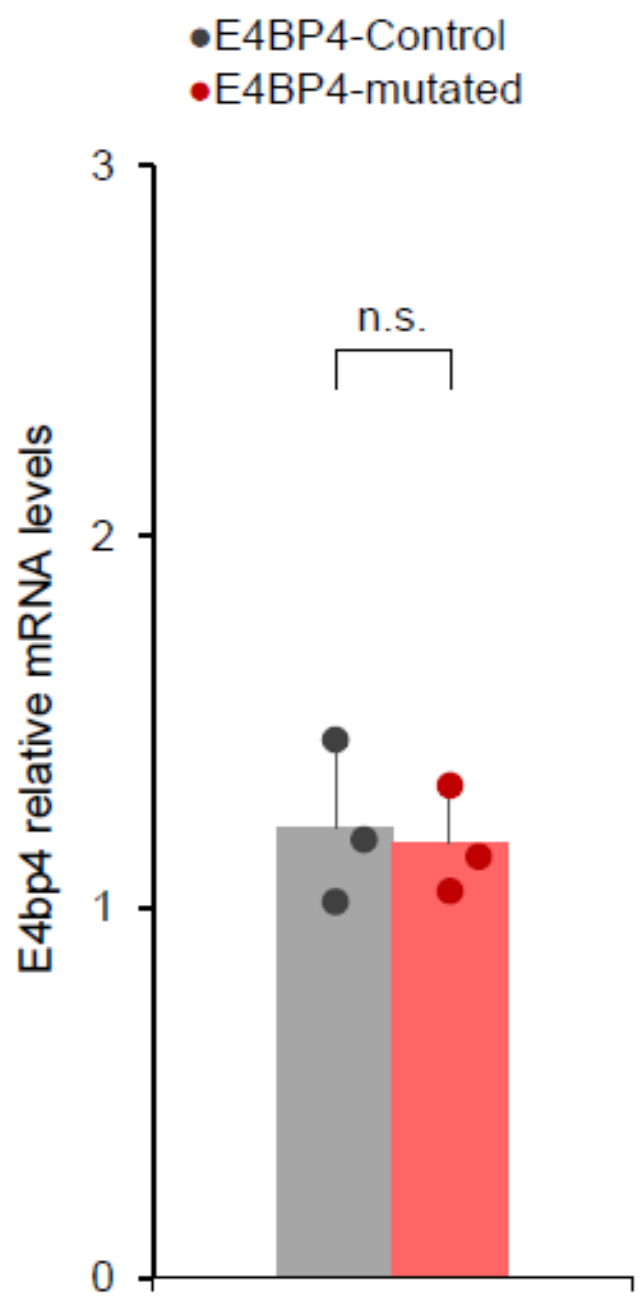

The new results are presented in **Appendix Fig. S1**

Reviewer #2 comment: The evidence of mitochondrial fragmentation is not convincing. In the reviewer's opinion, Figures 3E, 3G, 4F, and 5M demonstrated a decrease in mitochondrial quantity, but not fragmentation.

Response: We thank the reviewer for this observation. We have already addressed the comments from reviewer #1 (above) regarding Figures 3E, 3G and 4F related to measurements of mitochondria fragmentation. To strengthen the interpretation of these results, we have also performed a quantitative analysis of mitochondrial morphology parameters associated with Figure 5M. Please note that in the revised manuscript, **Figure 5M** has been renumbered as **Figure 6F**.

Specifically, we measured:

- **Mitochondrial volume (µm³):** reflecting overall mitochondrial size.
- **Surface area (µm²):** reflecting membrane expansion.
- **Sphericity index:** indicating morphological rounding, which increases with fragmentation.
- **Number of branches and branch junctions per mitochondrion:** reflecting mitochondrial networking and fusion.

As shown in the new analysis **(Figure EV4D)**, palmitate treatment significantly reduced mitochondrial volume, surface area, and branching, while increasing sphericity, consistent with enhanced mitochondrial fragmentation in control cells. Notably, these changes were significantly

blunted in the Cers6 enhancer edited cells (EΔ), supporting our conclusion that disruption of Cers6 protects against lipid overload–induced mitochondrial fragmentation**.** This text was added in the Results section of the revised manuscript (lines 487-491).

D

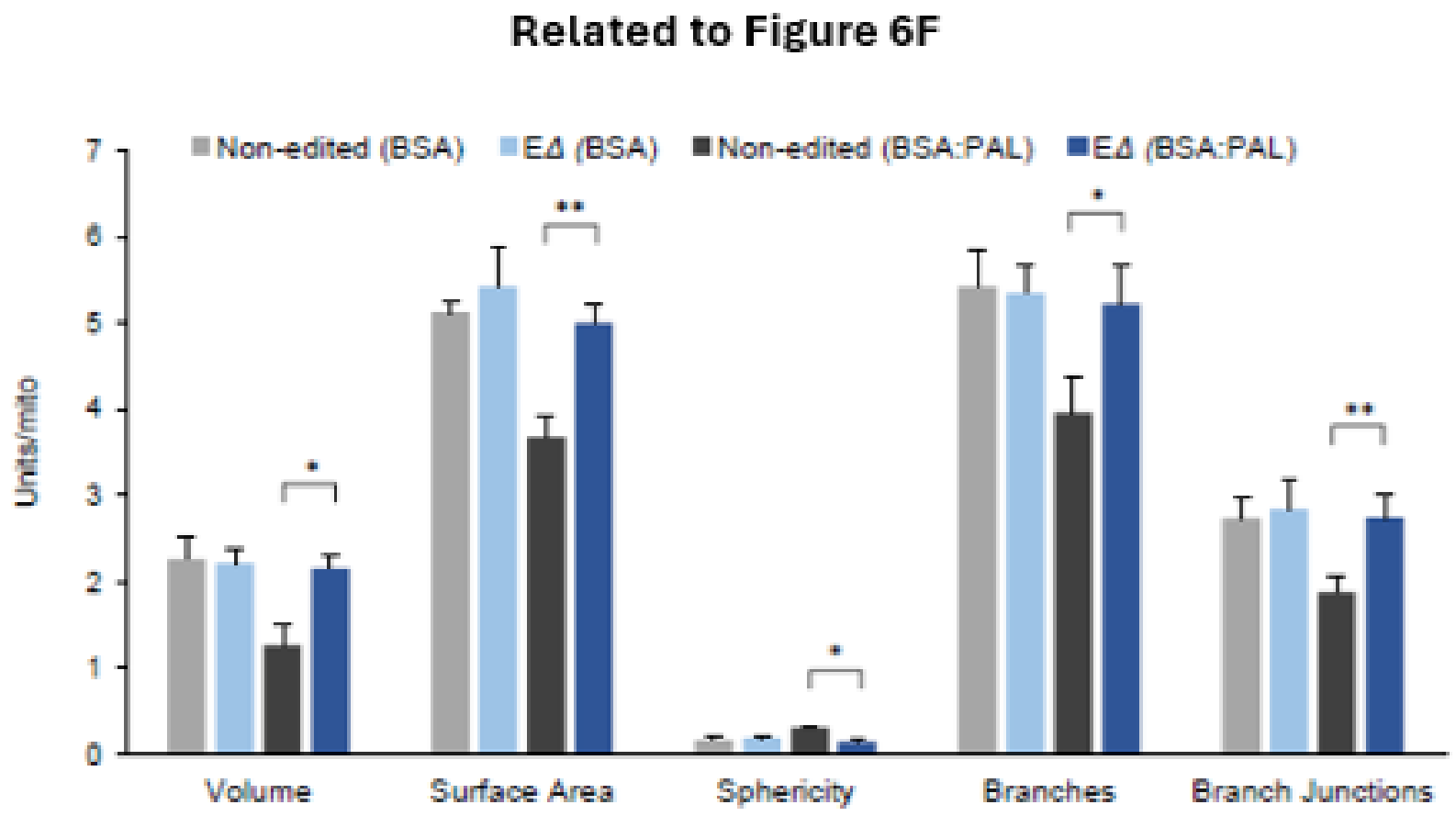

The new results are presented in **Figures EV4D**

Regarding the reviewer's understanding of a "decrease in mitochondrial quantity, but not fragmentation," we respectfully disagree. The analyses performed for Figures 3E, 3G, 4F, and 5M **(now Figure 6F)** clearly demonstrate that E4bp4 overexpression (E4bp4-OE) prevents lipid overload –induced mitochondrial fragmentation.

In relation to mitochondrial quantity, our data do not support differences in mitochondrial biogenesis between groups. Specifically, the expression of thermogenic and mitochondrial biogenesis genes **(Figure EV2G)** as well as the mitochondrial-to-nuclear DNA ratio **(Figure EV3D)** showed no significant changes, indicating that mitochondrial biogenesis is not altered.

Alternatively, it is possible that E4bp4 prevents mitophagy, as our results of Figure 3H **(now Figure EV5)** show that E4bp4-OE protects against lipid overload-induced mitochondrial depolarization. In this regard, previous studies have demonstrated that fragmented and depolarized mitochondria are targeted for degradation through mitophagy (DOI: 10.2337/db07-1781; DOI: 10.1074/jbc.M111.242412). While this explanation is consistent with our findings, we acknowledge that it remains speculative at this stage and, although interesting, is beyond the scope of the current study.

Reviewer #2 comment: It is confusing whether the association shown in Figure 1C is a positive or an inverse association.

Response: We thank the reviewer for pointing out this source of confusion. **Figure 1C** represents common variant associations for E4BP4, where the y-axis indicates the strength of association (-log10 p-value) rather than the direction (positive or inverse) of the effect. We have clarified this in the revised manuscript to avoid misinterpretation (lines 157-161). The associations indicate that

genetic variants in E4bp4 are positively linked with anthropometric traits such as weight, BMI, and waist-hip ratio.

FINAL REVISION

Reviewer #1 comment:
Figures B: Sample size of EE experiments is too low to draw any meaningful conclusions or to know for certain if the data are reproducible. Small sample sizes, likely coming from one litter and one batch of AAV are prone to type I error.

Response: We thank the reviewer for this important concern and agree that adequate sample size and reproducibility are essential for interpretation of energy expenditure (EE) data. To address this, we repeated the EE experiments using larger cohorts (n=8/group) of mice derived from independent litters and distinct AAV preparations. As expected, E4BP4 gain-of-function led to higher E4bp4 mRNA levels than in control. Moreover, our new data confirmed that E4BP4 overexpression increased EE after adjustment for total body mass, as determined by ANCOVA analysis (mass effect p = 0.067; group effect p = 0.017). In parallel, we complemented the *in vivo* EE measurements with independent ex vivo mitochondrial respiration assays in BAT, which demonstrated significantly higher oxygen consumption rates across multiple substrates in E4BP4-overexpressing mice compared with controls. The concordance between increased whole-body EE and enhanced tissue-level mitochondrial oxidative capacity across independent cohorts supports the robustness and reproducibility of the phenotype. We have updated the manuscript to include these additional data.

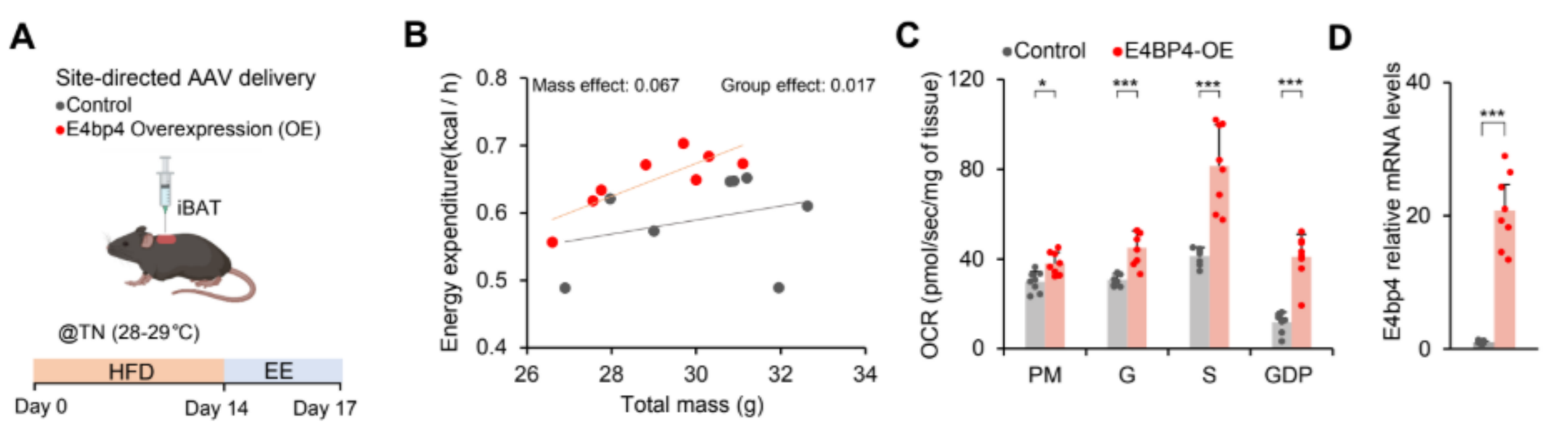

The new results are presented in **Figures 2A - 2D**.

Finally, we have updated in the manuscript the data regarding the body mass **(Figure EV2A)** and tissue mass **(Figure EV2B)** generated during this revision, which are consistent with previous findings.

Reviewer #1 comment:
Figure 3I: Why do cells (none of the groups) show no response to NE stimulation? Please clarify or provide potential mechanistic insight. Perhaps the cells were not differentiated well.

Response: We thank the reviewer for this important point. To clarify the limited NE response observed in the original experiment, we repeated the oxygen consumption assays comparing control and E4BP4-OE brown adipocytes under both BSA (vehicle) or BSA/palmitate (BSA:PAL) pretreatments. Our data clearly demonstrate that NE responsiveness was mainly influenced by lipid exposure rather than differentiation status or any other potential reason. Specifically, after short-term (6h) or long-term (24h) BSA pretreatment, both control and E4BP4-OE cells retained NE-stimulated OCR, indicating adequate differentiation and functional β-adrenergic signaling **(Figure 3H, Figure 3I, Table EV1)**. In contrast, short-term (6h) or long-term (24h) palmitate exposure markedly blunted NE-stimulated OCR in both control and E4BP4-OE brown adipocytes **(Figure 3H, Figure 3I, Table EV1).** Importantly, E4BP4-OE cells exhibited significantly higher NE-stimulated OCR after long-term (24 h) palmitate pretreatment compared with control cells **(Figure 3H, Figure 3I, Table EV1)**, indicating that NE-stimulated OCR is preserved in the E4BP4-OE group despite prolonged lipid overload.

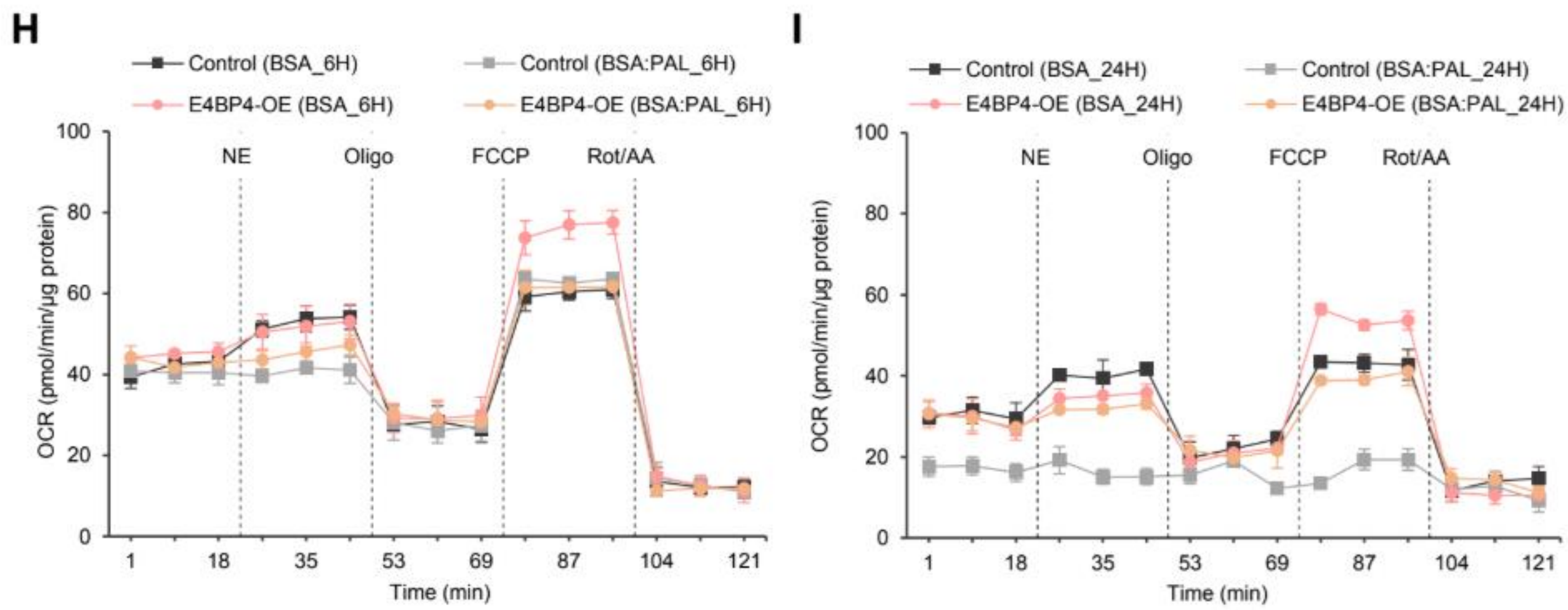

The new results are presented in **Figures 3H, 3I**

We have revised the manuscript to clarify this point (lines 325-341).

Reviewer #1 comment: Figures 3I vs 5N. There is a striking discrepancy between these panels. In both, cells were treated with palmitate for 6 h, yet the NE and CCCP responses differ significantly. Are these the same cell types and conditions? Please reconcile the differences.

Response: We would like to clarify that Figure 3I **(now Figure 3H, Figure 3I, Table EV1)** and Figure 5N **(now Figure 6G, Figure 6H, Table EV1)** represent different experimental systems: Figures 3H and 3I shows data from primary brown adipocytes with E4bp4 transgene overexpression, whereas Figures 6G and 6H shows data from immortalized brown adipocytes with Cas9-mediated mutation of a 65 kb Cers6 enhancer site. Given the distinct cell types and genetic manipulations, a direct comparison between these two panels is not appropriate. Nevertheless, we agree that confirming the consistency of the phenotype is important. Across both genetic systems, short-term palmitate exposure (6 h) was associated with preserved basal respiratory capacity, although NE-stimulated and FCCP-stimulated OCR response varied

depending on the genetic context, indicating that mitochondrial oxidative capacity remains largely intact at early stages of lipid stress **(Figures 3H and 6G)**. In contrast, prolonged palmitate exposure (24 h) consistently revealed a pronounced suppression of basal and maximal respiratory capacity in control **(Figure 3I)** and non-edited (**Figure 6H)** brown adipocytes, reflecting mitochondrial oxidative dysfunction. These defects were mitigated in genetically protected conditions, including E4BP4-overexpressing brown adipocytes and cells harboring deletion of the upstream Cers6 enhancer, both of which maintained robust basal and maximal OCR under chronic lipid overload **(Figures 3I and 6H)**. Notably, preservation of NE-stimulated OCR at 24 h was observed specifically in the E4BP4 overexpression model **(Figure 3I)**, indicating differential protection of adrenergic responsiveness depending on the mode of genetic intervention.

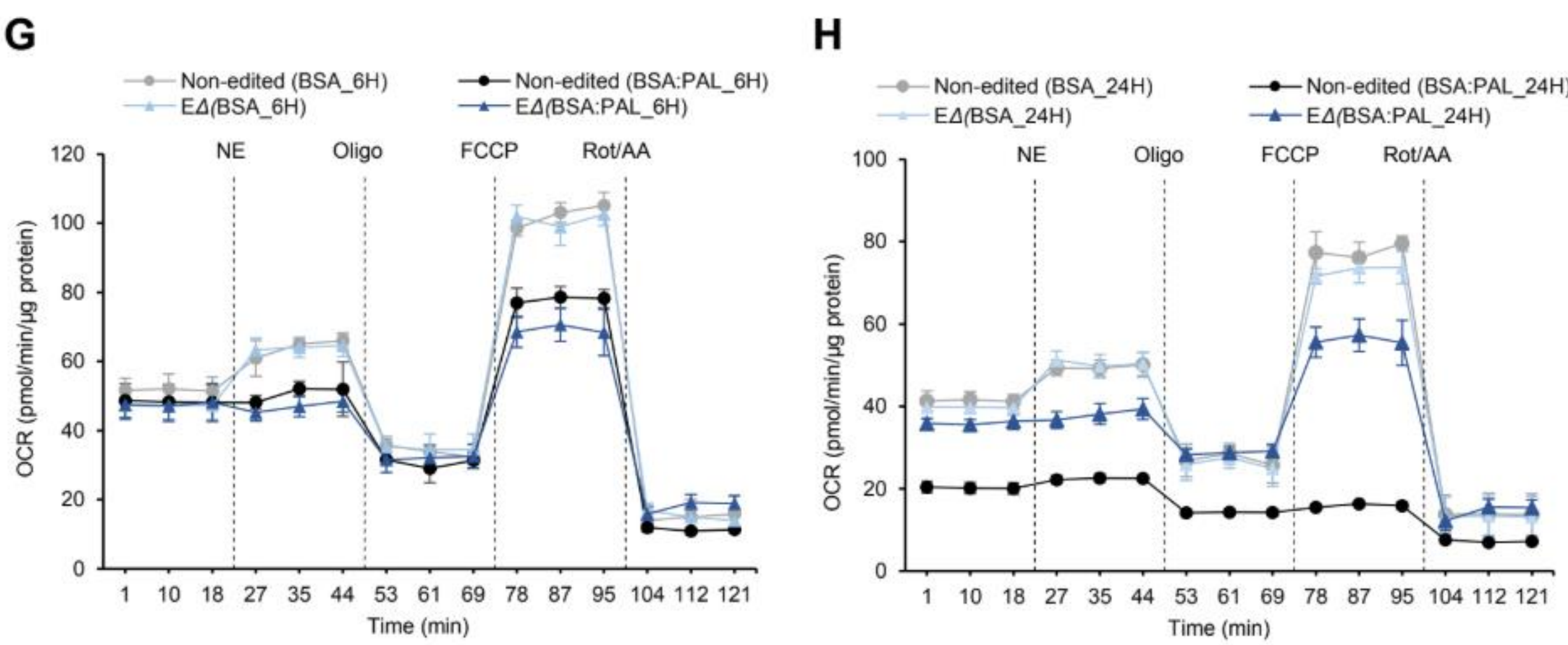

The new results are presented in **Figures 6G, 6H**

We have revised the manuscript to clarify this point (lines 325-341 and 491-503).

Reviewer #2 comment: A key experiment is missing: does adding C16:0 block the mitochondrial benefits of E4BP4-OE?

Response: We thank the reviewer for this excellent suggestion and agree that it is essential to test whether restoration of C16:0 ceramide biosynthesis interferes with the mitochondrial benefits of E4BP4 overexpression. To address this, we performed a “rescue” experiment by co-overexpressing E4BP4 and Cers6 in brown adipocytes, using E4BP4 overexpression alone as the control. Importantly, the Cers6 transgene was driven by a CMV promoter, rendering its expression insensitive to E4BP4-mediated repression. Gene expression analyses confirmed robust induction of Cers6 mRNA levels in the co-overexpression condition **(Figure 5H)**. Restoration of Cers6 expression was sufficient to reverse the mitochondrial phenotype conferred by E4BP4-OE. Quantitative morphological analyses revealed pronounced mitochondrial

fragmentation, including reduced mitochondrial volume and surface area, increased sphericity, and decreased branching, compared with E4BP4-OE cells **(Figure 5I, Figure EV4C)**. Consistent with these structural changes, co-overexpression of Cers6 markedly impaired cellular respiration, with significant reductions in ATP-linked and FCCP-stimulated maximal OCR, indicating loss of mitochondrial oxidative capacity **(Figure 5J)**. Collectively, these findings provide functional evidence that repression of Cers6-dependent ceramide synthesis is a critical component of the mitochondrial protection afforded by E4BP4 overexpression and that restoration of this pathway counteracts the mitochondrial benefits associated with E4BP4 gain-of-function.

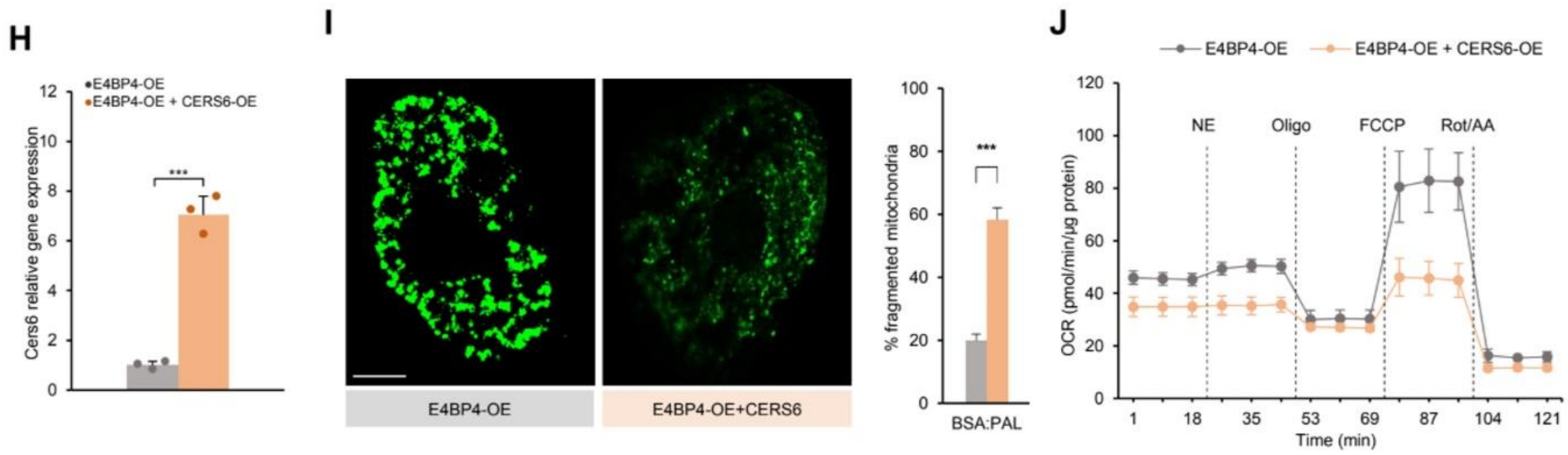

The new results are presented in **Figures 5H - 5J**

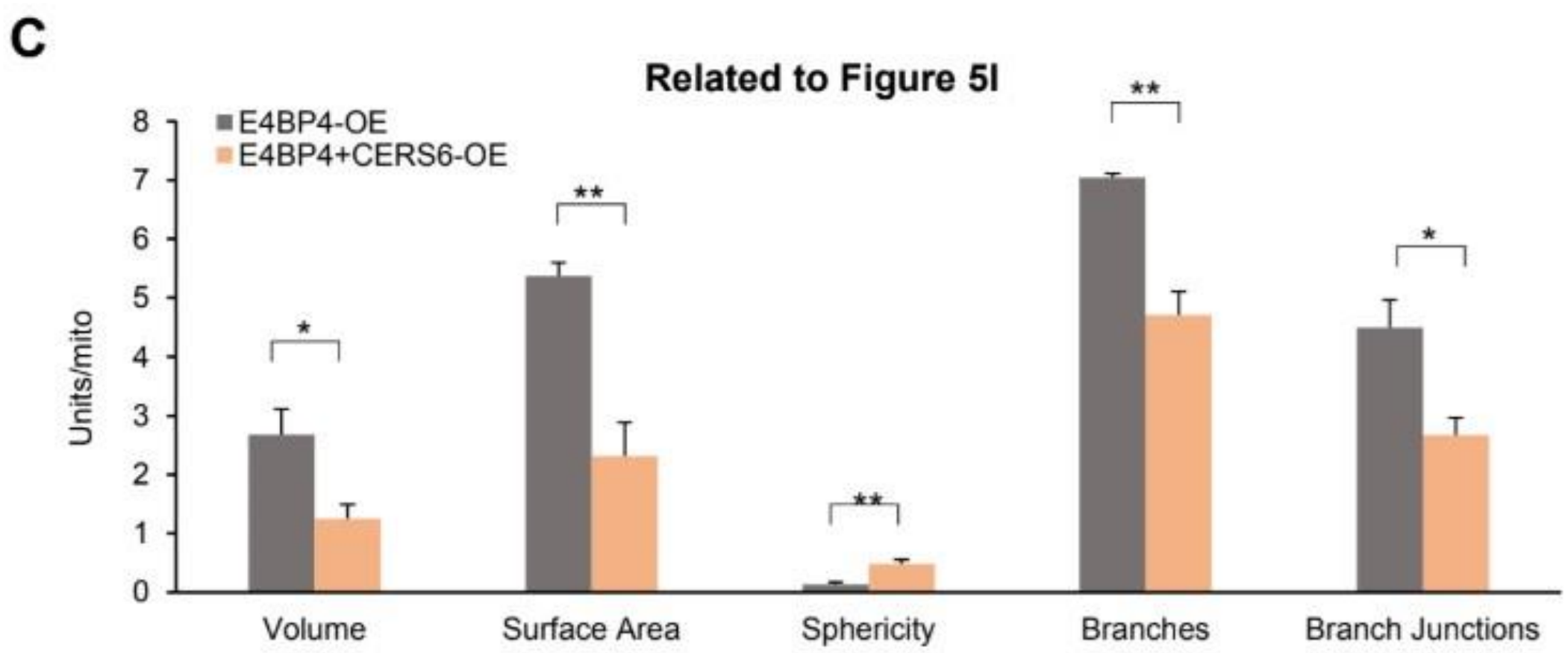

The new results are presented in **Figure EV4C**

We have revised the manuscript to clarify this point (lines 414-437).

Reviewer #2 comment: Whether PRDM16-OE mimics the effects of E4BP4 to induce p-Drp1 is not shown.

Response: We thank the reviewer for this valuable suggestion. We agree that testing whether PRDM16 overexpression mimics the effects of E4BP4 on p-Drp1 is important to strengthen the mechanistic link between these transcription factors in terms of regulation of mitochondrial fragmentation. Our data demonstrate that despite both PRDM16-OE and E4BP4-OE reduce

Cers6 expression and C16:0 ceramide levels, the reduction of ceramide abundance alone is not sufficient to suppress Drp1/Mff-dependent mitochondrial fission. E4BP4 gain-of-function uniquely increases inhibitory Drp1(S637) phosphorylation **(Figure 5G)**, consistent with functional disengagement of the fission machinery, whereas PRDM16-OE does not alter Drp1 phosphorylation despite lowering C16:0 ceramides **(Figure 7G)**. We therefore propose that PRDM16 primarily regulates ceramide availability, while E4BP4 acts at an additional downstream regulatory node to uncouple residual ceramide signaling from the Drp1 – Mff axis, thereby preventing mitochondrial fragmentation.

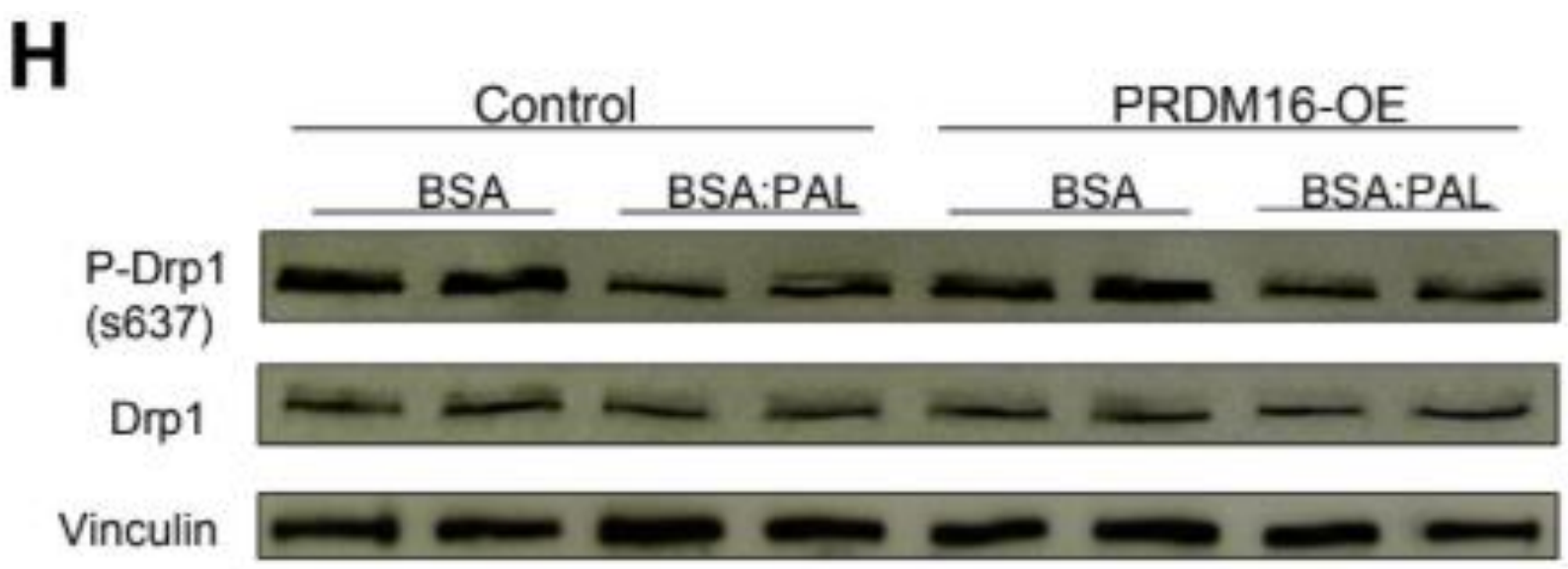

The new results are presented in **Figure 7G**

We have revised the manuscript to clarify this point (lines 531-535 and 614-618).

5. Description of analyses that authors prefer not to carry out

Please include a point-by-point response explaining why some of the requested data or additional analyses might not be necessary or cannot be provided within the scope of a revision. This can be due to time or resource limitations or in case of disagreement about the necessity of such additional data given the scope of the study. Please leave empty if not applicable.

Reviewer #2 comment:
It would be worthwhile to investigate whether in vivo knockdown of E4BP4 blunts the Cers6-suppressing effects of PRDM16-OE.

Response: We agree that assessing in vivo loss-of-function of E4bp4 in the context of Prdm16 overexpression would be highly informative. At present, this experiment is technically not feasible, as it would require the generation and characterization of complex *in vivo* models beyond the scope of the current study. Nevertheless, we are actively considering this as a future direction. In the meantime, we believe that the *in vitro* experiments in brown adipocytes provided here are sufficient to establish the mechanistic relationship between E4BP4 and PRDM16 in the regulation of Cers6 expression.

Reviewer #2 comment: Whether E4BP4-OE affects cold tolerance in mice is now shown.

Response: We thank the reviewer for this thoughtful comment. In our study, we performed an iBAT-specific E4bp4 gain-of-function assay because we observed a downregulation of E4bp4 expression in the context of obesity. The rationale for this approach was to rescue E4bp4 expression in iBAT and thereby evaluate its systemic and mechanistic effects under obesogenic conditions. We recognize that a gain-of-function assay during cold challenge would further enhance E4bp4 expression and, while interesting, this would more directly address the role of E4bp4 in thermogenic regulation rather than in obesity-related metabolic dysfunction. For this reason, we believe that a detailed investigation of E4bp4 in cold-induced thermogenesis is an important but separate question that lies beyond the scope of the current study.

1st Revision - Editorial Decision 23rd Apr 2026

Dear Prof. Sponton

Thank you for the submission of your revised manuscript to EMBO reports. We have now received the full set of referee reports that is copied below.

As you will see, all referees are very positive about the study. We believe that only textual changes will be required to address any remaining concerns. Please provide a PBP response indicating how you have addressed the remaining concerns.

From the editorial side, there are also a few things that we need before we can proceed with the official acceptance of your study.

- As a standard procedure, we edit the abstract of manuscripts to make them more accessible to a general readership. Please find the suggested version below my signature, which has been edited to maintain present tense.

-Please rename the "Summary" section to "Abstract".

- Please rename the "Resource Availability" section to "Data Availability".

- Please remove the "Lead Contact" and "Material Availability" from the manuscript.

- Please remove the "Supplementary Information" section from the manuscript as well as the "Expanded View Figures" section immediately above it.

- Please arrange the manuscript sections in the following order: Title page - Abstract & Keywords - Introduction - Results - Discussion - Methods - Data Availability - Acknowledgments - Disclosure Statement & Competing Interests - References - Figure Legends - (Main Tables with legends if applicable) - Expanded View Figure Legends.

- Please declare the use of AI in the Methods section.

- As we are switching from a free-text author contribution statement towards a more formal statement based on Contributor Role Taxonomy (CRediT) terms, please remove the present Author Contribution section and instead specify each author's contribution(s) directly in the Author Information page of our submission system during upload of the final manuscript. See https://casrai.org/credit/ for more information.

- Please adjust the format of the reference list and of the in-text citations according to EMBO Journal format (alphabetical order, author name et al + year.../up to 10 author names in the reference list before et al) Please refer to our Guide to Authors for additional information on EMBO Press reference format.

- We need you to complete and upload our dedicated author checklist (https://link.springer.com/journal/44319/submission-guidelines). Please insert information in the checklist that is also reflected in the manuscript. The completed author checklist will also be part of the Peer Review File.

- Please make sure to all relevant funding information in the manuscript, including the Acknowledgements section, is also entered into our submission system. The funding information in the manuscript needs to be congruent with the info entered into our online system.

- Please adjust the in-text callouts for individual figures and figure panels. For instance, "supplementary" should not be used as nomenclature and "Data S2" is not a correct callout.

- Table S1, S2, and S4 have been reorganized into Datasets EV1-EV3. Tables S3 and S5 have been updated to Tables EV1-EV2. Please update the callouts in the manuscript accordingly. Please provide each table legend in its corresponding file. The legends of datasets can be provided in a separate sheet/tab.

- Please remove dataset, EV table and video legends from the Appendix file and place in their respective table/dataset files.

- Please remove the uncropped blots from the Appendix file and place in their respective source data folders.

- Please consider whether the remaining 1 figure in the Appendix file should be turned into an EV figure.

- Please update the callouts throughout the manuscript for the uploaded movie to "Movie EV1". Please remove the legend from the manuscript and Appendix and provide in a separate text file that is zipped with the movie file and uploaded as Movie EV1.

- Please note that EMBO press papers are accompanied online by:
A) a short (2 sentences) summary of the findings and their significance,
B) 2-5 short bullet points highlighting the key results, and
C) a synopsis image in .jpg or .png format that is exactly 550 pixels wide and 300 pixels high. Please note that the text needs to be legible at the final size. Please upload this information along with your revised manuscript (the text for A and B should be provided in one separate Word file uploaded as Cover Art/Synopsis Image).

- Please provide the completed Reagent and Tools Table and upload it as an individual file. For more information, please check https://www.embopress.org/page/journal/14602075/authorguide#structuredmethods and download the template for Reagent Table

- Source Data files have been re-organized to match the Source Data checklist. Please ensure that the blots are removed from the Appendix file to their corresponding Source Data folders.

- Please ensure that the URL for the large deposited Source Data is in the Data availability statement.

- Please note this report from our Figure Check:
The resolution of the submitted figures is too low for blot and microscopy images. This reduction in resolution is commonly caused by converting original 16-bit TIFF files to RGB format for publication. While this is not inherently problematic, it can raise concerns about image integrity for critical readers.
To avoid any misunderstanding and to meet EMBO Press standards, we kindly ask that you:
* Resubmit the complete figure set at the captured original data resolution.
* Apply the same resolution standards to the blot source data files, which are also currently below the required quality threshold. Please upload the blot source data files as .tiff or pdf. Please do not use PowerPoint.

-Please ensure that all publicly available datasets re-analyzed for this manuscript, such as Li, Jing et al. 2023, receive a dataset citation. For formatting, please refer to https://link.springer.com/journal/44318/submission-guidelines#cms-Reference-guidelines.

- Please note that all graphs with n<5 should have individual data points plotted. Please doublecheck this is the case, particularly for figures 3E, 3G, 4F, 5G, 5I, EV3B, EV3C, EV3D, EV3F, EV4A, EV4B, EV4C, EV4D, EV5.

- Our production/data editors have asked you to clarify several points in the figure legends (see below). Please incorporate these changes in the manuscript and return the revised file with tracked changes with your final manuscript submission.
- Please define the annotated p values ****/***/**/* as well as provide the exact p-values for the same in the legend of figure EV5 as appropriate.
- Please note that the exact p values are not provided in the legends of figures 1D, F, G, I;2C, D, F, G, H, I, J, L; 3C, E, G; 4F, 5A, B, C, D, E, G, H, I; 6A, C, F; 7D-F; EV1 E-G; EV2 B, C, D, G; EV3 B, C, F, H, I; EV4 A-D.
- Please indicate the statistical test used for data analysis in the legend of figure EV5
- Please note that information related to n is missing in the legends of figures 3G, 5G, EV2 F, EV3 B, EV5
- Please note that the error bars are not defined in the legend of figure EV5.
- Please note that the dashed circles are not defined in the legend of figure 3G. This needs to be rectified.
- Please note that the red arrows are not defined in the legend of figure 3A. This needs to be rectified.

-Can you explain the apparent duplications in the attached source data files?

We look forward to seeing a final version of your manuscript as soon as possible.

Kurt Weir
Editor
EMBO Reports

Brown adipose tissue (BAT) counteracts obesity-related metabolic dysfunction through both thermogenic and non-thermogenic means. However, substantial evidence indicates that obesity negatively affects BAT mitochondrial morphology and oxidative capacity, impairing systemic energy homeostasis. Motivated by this apparent contradiction, we investigate the relationship between obesity and mitochondrial dynamics, as the underlying mechanisms remain incompletely understood. Here, we identify E4BP4 as a transcriptional repressor that prevents obesity-induced mitochondrial fragmentation and oxidative dysfunction by inhibiting ceramide synthesis in brown fat. Specifically, E4BP4 interacts with PRDM16 to repress Cers6 mRNA expression and consequently reduces C16:0 ceramide levels by binding to a 65 kb upstream enhancer region of the Cers6 gene. Notably, the preservation of mitochondrial integrity in BAT by E4BP4 gain-of-function improves systemic glucose homeostasis, independent of weight loss. Collectively, our findings establish E4BP4 as a molecular safeguard against obesity-induced mitochondrial fragmentation and oxidative dysfunction, primarily by suppressing ceramide synthesis in brown fat.

Referee #1:

This revision well addressed most of my previous concerns. One issue remains regarding the uncharacterized cold tolerance in the E4BP4-OE model. Although I agree that cold tolerance might be an independent measurement from diet-induced obesity, it is considered an important parameter of iBAT mitochondrial health.

Referee #2:

The authors have addressed my concerns

Editorial & Production Requirements (EMBO Reports)

EMBOR-2025-62856V2

- **As a standard procedure, we edit the abstract of manuscripts to make them more accessible to a general readership. Please find the suggested version below my signature, which has been edited to maintain present tense.**

R. The editorially edited version of the abstract has been incorporated into the manuscript.

- **Please rename the "Summary" section to "Abstract".**

R. The section has been renamed accordingly.

- **Please rename the "Resource Availability" section to "Data Availability".**

R. The section has been renamed accordingly.

- **Please remove the "Lead Contact" and "Material Availability" from the manuscript.**

R. These information have been removed.

- **Please remove the "Supplementary Information" section from the manuscript as well as the "Expanded View Figures" section immediately above it.**

R. Both sections have been removed.

- **Please arrange the manuscript sections in the following order. Title page - Abstract & Keywords - Introduction - Results - Discussion - Methods - Data Availability - Acknowledgments - Disclosure Statement & Competing Interests - References - Figure Legends - (Main Tables with legends if applicable) - Expanded View Figure Legends.**

R. The manuscript has been reorganized to follow the requested structure.

- **Please declare the use of AI in the Methods section.**

R. A statement declaring the use of AI has been included in the Methods section.

- **As we are switching from a free-text author contribution statement towards a more formal statement based on Contributor Role Taxonomy (CRediT) terms, please remove the present Author Contribution section and instead specify each author's contribution(s) directly in the Author Information page of our submission system during upload of the final manuscript. See https://casrai.org/credit/ for more information.**

R. The Author Contributions section has been removed, and contributions were specified in the submission system in accordance with CRediT guidelines.

- **Please adjust the format of the reference list and of the in-text citations according to EMBO Journal format (alphabetical order, author name et al + year.../up to 10 author names in the reference list before et al) Please refer to our Guide to Authors for additional information on EMBO Press reference format.**
R. The reference list and in-text citations have been reformatted accordingly.

- **We need you to complete and upload our dedicated author checklist (https://link.springer.com/journal/44319/submission-guidelines). Please insert information in the checklist that is also reflected in the manuscript. The completed author checklist will also be part of the Peer Review File**
R. The author checklist has been completed and was uploaded with the revised manuscript.

- **Please make sure to all relevant funding information in the manuscript, including the Acknowledgements section, is also entered into our submission system. The funding information in the manuscript needs to be congruent with the info entered into our online system..**
R. Funding information has been reviewed and were consistently reported in both the manuscript and submission system.

- **Please adjust the in-text callouts for individual figures and figure panels. For instance, "supplementary" should not be used as nomenclature and "Data S2" is not a correct callout.**
R. All figure and panel callouts have been corrected.

- Table S1, S2, and S4 have been reorganized into Datasets EV1-EV3. Tables S3 and S5 have been updated to Tables EV1-EV2. Please update the callouts in the manuscript accordingly.
R. The tables have been reorganized as requested, and all corresponding callouts have been updated.

- **Please provide each table legend in its corresponding file. The legends of datasets can be provided in a separate sheet/tab.**
R. All table legends have been provided within their respective files.

- **Please remove dataset, EV table and video legends from the Appendix file and place in their respective table/dataset files.**
R. These legends have been removed from the Appendix and relocated to the appropriate files.

- **Please remove the uncropped blots from the Appendix file and place in their respective source data folders.**
R. Uncropped blots have been removed from the Appendix and placed in the appropriate Source Data folders.

- **Please consider whether the remaining 1 figure in the Appendix file should be turned into an EV figure.**
R. The remaining figure has been converted into an Expanded View figure (EV6).

- **Please update the callouts throughout the manuscript for the uploaded movie to "Movie EV1". Please remove the legend from the manuscript and Appendix and provide in a separate text file that is zipped with the movie file and uploaded as Movie EV1.**
R. The movie has been renamed “Movie EV1”, and its legend has been provided in a separate file.

- **Please note that EMBO press papers are accompanied online by: A) a short (2 sentences) summary of the findings and their significance, B) 2-5 short bullet points highlighting the key results, and C) a synopsis image in .jpg or .png format that is exactly 550 pixels wide and 300 pixels high. Please note that the text needs to be legible at the final size. Please upload this information along with your revised manuscript (the text for A and B should be provided in one separate Word file uploaded as Cover Art/Synopsis Image).**
R. These materials were provided in a separate file as requested.

- **Please provide the completed Reagent and Tools Table and upload it as an individual file.**
R. The table has been completed and will be uploaded as an individual file.

- **Source Data files have been re-organized to match the Source Data checklist. Please ensure that the blots are removed from the Appendix file to their corresponding Source Data folders Appendix.**

R. Source Data files have been reorganized accordingly

- **Please ensure the URL for large deposited Source Data is in the Data Availability statement.**
R. The URL has been included

- **Please note this report from our Figure Check: The resolution of the submitted figures is too low for blot and microscopy images. This reduction in resolution is commonly caused by converting original 16-bit TIFF files to RGB format for publication. While this is not inherently problematic, it can raise concerns about image integrity for critical readers. To avoid any misunderstanding and to meet EMBO Press standards, we kindly ask that you: * Resubmit the complete figure set at the captured original data resolution. * Apply the same resolution standards to the blot source data files, which are also currently below the required quality threshold. Please upload the blot source data files as .tiff or pdf. Please do not use PowerPoint.**

R. Regarding the concerns about the resolution of images, we have updated the figures using TIFF format to prevent any loss of quality; furthermore, we have provided the original raw images in the "Source Data". While we acknowledge that the resolution is not at a high-definition level, this is a direct limitation of the technical specifications and maximum output of our blot imaging system imageQuant LAS4000 (GE), and the provided files represent the highest quality achievable with our current laboratory equipment.

- **Please ensure that all publicly available datasets re-analyzed for this manuscript, such as Li, Jing et al. 2023, receive a dataset citation. For formatting, please refer to https://link.springer.com/journal/44318/submission-guidelines#cms-Reference-guidelines.**

R. Dataset citations have been added, including appropriate references and links.

- **Please note that all graphs with n<5 should have individual data points plotted. Please doublecheck this is the case, particularly for figures 3E, 3G, 4F, 5G, 5I**

R. All relevant graphs have been updated to include individual data points.

- **Please note that all graphs with n<5 should have individual data points plotted. Please doublecheck this is the case, particularly for figures EV3B, EV3C, EV3D, EV3F, EV4A, EV4B, EV4C, EV4D, EV5.**

R. All relevant EV graphs have been updated accordingly.

- **Our production/data editors have asked you to clarify several points in the figure legends (see below). Please incorporate these changes in the manuscript and return the revised file with tracked changes with your final manuscript submission.**

R. All requested clarifications have been incorporated into the figure legends.

- **Please define the annotated p values //**/ as well as provide the exact p-values for the same in the legend of figure EV5 as appropriate).**

R. Annotated p-values have been defined, and exact values have been included.

- **Please note that the exact p values are not provided in the legends of figures 1D, F, G, I; 2C, D, F, G, H, I, J, L; 3C, E, G; 4F, 5A, B, C, D, E, G, H, I; 6A, C, F; 7D-F; EV1 E-G; EV2 B, C, D, G; EV3 B, C, F, H, I; EV4 A-D.**

R. Exact p-values have been added to all specified figure legends.

- **- Please indicate the statistical test used for data analysis in the legend of figure EV5.**

R. The statistical test has been specified in the legend.

- **Please note that information related to n is missing in the legends of figures 3G, 5G, EV2 F, EV3 B, EV5.**

R. Sample size (n) has been added to all figure legends.

- **Please note that the error bars are not defined in the legend of figure EV5.**

R. Error bars have been clearly defined in the legend.

- **Please note that the dashed circles are not defined in the legend of figure 3G. This needs to be rectified.**

R. Dashed circles are now defined in the figure legend.

- **Please note that the red arrows are not defined in the legend of figure 3A. This needs to be rectified.**

R. Red arrows are now defined in the figure legend.

- **Can you explain the apparent duplications in the attached source data files?**

R. We have identified that the apparent duplications in the source data files occurred due to unnecessary replication of values in two instances: (i) Figure 1F (Nfil3 mRNA levels in the TN control group) and (ii) Figure 7D (36b4 mRNA levels, used as the housekeeping gene). These duplications were inadvertent and do not reflect independent measurements. We have now corrected both source data files to remove these redundancies. In addition, we have adjusted Figure 1F accordingly to ensure full consistency between the source data and the plotted values.

- **Before our data integrity team can check the figure issues, can you please resolve the remaining formatting points:**

Remove the following sentences from the Data Availability since this section is reserved only for the deposited data:

- **This paper does not report original code.**
- **Any additional information required to reanalyze the data reported in this work paper is available from the lead contact upon request.**

R. These information have been removed accordingly.

- **We need a clearly labeled Methods section, please provide it or perhaps rename EXPERIMENTAL MODEL AND STUDY PARTICIPANT 670 DETAILS to METHODS?**

R. This change has been performed accordingly

- **We need you to complete and upload our dedicated author checklist (https://link.springer.com/journal/44319/submission-guidelines). Please**

insert information in the checklist that is also reflected in the manuscript. The completed author checklist will also be part of the Peer Review File. The file is attached to this email for your convenience.
R. Author checklist has been added accordingly

- **Table EV2 is missing a legend, please provide it in the table file.**

R. Table EV2 legend has been added accordingly

2nd Revision - Editorial Decision 26th May 2026

Prof. Carlos Sponton
Ribeirão Preto Medical School, Universidade de São Paulo (FMRP-USP), Ribeirão Preto, Brazil
Physiology
Brazil

Dear Prof. Sponton,

I am pleased to inform you that your manuscript has been accepted for publication in EMBO reports. Your manuscript will be processed for publication by EMBO Press. It will be copy edited and you will receive page proofs prior to publication. Please note that you will be contacted by Springer Nature Author Services to complete licensing and payment information.

You may qualify for financial assistance for your publication charges - either via a Springer Nature fully open access agreement or an EMBO initiative. Check your eligibility: https://link.springer.com/journal/44319/how-to-publish-with-us

Should you be planning a Press Release on your article, please notify the production team via this form in order to coordinate publication and release dates: https://forms.cloud.microsoft/e/eH4A8JVP6g

If you have any questions, please do not hesitate to contact the Editorial Office. Thank you for your contribution to EMBO Reports.

Yours sincerely,

Kurt Weir
Editor
EMBO Reports

>>> Please note that it is EMBO Reports policy for the transcript of the editorial process (containing referee reports and your response letter) to be published as an online supplement to each paper. If you do NOT want this, you will need to inform the Editorial Office via email immediately. More information is available here: https://link.springer.com/partners/embo-press/editorial-policies#Peer%20review

EMBO Press Author Checklist

Corresponding Author Name: Carlos Henrique Sponton
Journal Submitted to: EMBO Reports
Manuscript Number: EMBOR-2025-62856V3

USEFUL LINKS FOR COMPLETING THIS FORM
The EMBO Journal - Author Guidelines
EMBO Reports - Author Guidelines
Molecular Systems Biology - Author Guidelines
EMBO Molecular Medicine - Author Guidelines

Reporting Checklist for Life Science Articles (updated January
This checklist is adapted from Materials Design Analysis Reporting (MDAR) Checklist for Authors. MDAR establishes a minimum set of requirements in transparent reporting in the life sciences (see Statement of Task: 10.31222/osf.io/9sm4x). Please follow the journal's guidelines in preparing your
Please note that a copy of this checklist will be published alongside your article.

Abridged guidelines for figures
1. Data
The data shown in figures should satisfy the following conditions:

- the data were obtained and processed according to the field's best practice and are presented to reflect the results of the experiments in an accurate and unbiased manner.
- ideally, figure panels should include only measurements that are directly comparable to each other and obtained with the same assay.
- plots include clearly labeled error bars for independent experiments and sample sizes. Unless justified, error bars should not be shown for technical
- if n<5, the individual data points from each experiment should be plotted. Any statistical test employed should be justified.
- Source Data should be included to report the data underlying figures according to the guidelines set out in the authorship guidelines on Data

2. Captions
Each figure caption should contain the following information, for each panel where they are relevant:

- a specification of the experimental system investigated (eg cell line, species name).
- the assay(s) and method(s) used to carry out the reported observations and measurements.
- an explicit mention of the biological and chemical entity(ies) that are being measured.
- an explicit mention of the biological and chemical entity(ies) that are altered/varied/perturbed in a controlled manner.
- the exact sample size (n) for each experimental group/condition, given as a number, not a range;
- a description of the sample collection allowing the reader to understand whether the samples represent technical or biological replicates (including how many animals, litters, cultures, etc.).
- a statement of how many times the experiment shown was independently replicated in the laboratory.
- definitions of statistical methods and measures:
  - common tests, such as t-test (please specify whether paired vs. unpaired), simple χ^2 tests, Wilcoxon and Mann-Whitney tests, can be unambiguously identified by name only, but more complex techniques should be described in the methods section;
  - are tests one-sided or two-sided?
  - are there adjustments for multiple comparisons?
  - exact statistical test results, e.g., P values = x but not P values < x;
  - definition of 'center values' as median or average;
  - definition of error bars as s.d. or s.e.m.

Please complete ALL of the questions below.
Select "Not Applicable" only when the requested information is not relevant for your study.

Materials

Newly Created Materials	Information included in the manuscript?	In which section is the information available? (Reagents and Tools Table, Materials and Methods, Figures, Data Availability Section)
New materials and reagents need to be available; do any restrictions apply?	Not Applicable	

Antibodies	Information included in the manuscript?	In which section is the information available? (Reagents and Tools Table, Materials and Methods, Figures, Data Availability Section)
For **antibodies** provide the following information:<br>- Commercial antibodies: RRID (if possible) or supplier name, catalogue number and or/clone number<br>- Non-commercial: RRID or citation	Yes	Reagents and tools table

DNA and RNA sequences	Information included in the manuscript?	In which section is the information available? (Reagents and Tools Table, Materials and Methods, Figures, Data Availability Section)
**Short novel DNA or RNA including primers, probes**: provide the sequences.	Yes	Table EV2

Cell materials	Information included in the manuscript?	In which section is the information available? (Reagents and Tools Table, Materials and Methods, Figures, Data Availability Section)
**Cell lines:** Provide species information, strain. Provide accession number in repository **OR** supplier name, catalog number, clone number, and/**OR** RRID.	Yes	Reagents and tools table
**Primary cultures:** Provide species, strain, sex of origin, genetic modification status.	Yes	Reagents and tools table
Report if the cell lines were recently **authenticated** (e.g., by STR profiling) and tested for mycoplasma contamination.	Not Applicable	

Experimental animals	Information included in the manuscript?	In which section is the information available? (Reagents and Tools Table, Materials and Methods, Figures, Data Availability Section)
**Laboratory animals or Model organisms:** Provide species, strain, sex, age, genetic modification status. Provide accession number in repository **OR** supplier name, catalog number, clone number, **OR** RRID.	Yes	Reagents and tools table
**Animal observed in or captured from the field:** Provide species, sex, and age where possible.	Not Applicable	
Please detail **housing and husbandry conditions**.	Yes	Methods (Mouse husbandry)

Plants and microbes	Information included in the manuscript?	In which section is the information available? (Reagents and Tools Table, Materials and Methods, Figures, Data Availability Section)
**Plants:** provide species and strain, ecotype and cultivar where relevant, unique accession number if available, and source (including location for collected wild specimens).	Not Applicable	
**Microbes:** provide species and strain, unique accession number if available, and source.	Yes	Reagents and tools table

Human research participants	Information included in the manuscript?	In which section is the information available? (Reagents and Tools Table, Materials and Methods, Figures, Data Availability Section)
If collected and within the bounds of privacy constraints report on age, sex and gender or ethnicity for all study participants.	Not Applicable	

Core facilities	Information included in the manuscript?	In which section is the information available? (Reagents and Tools Table, Materials and Methods, Figures, Data Availability Section)
If your work benefited from core facilities, was their service mentioned in the acknowledgments section?	Yes	Acknowledgments

Design

Study protocol	Information included in the manuscript?	In which section is the information available? (Reagents and Tools Table, Materials and Methods, Figures, Data Availability Section)
If study protocol has been **pre-registered, provide DOI in the manuscript**. For clinical trials, provide the trial registration number **OR** cite DOI.	Not Applicable	
Report the **clinical trial registration number** (at ClinicalTrials.gov or equivalent), where applicable.	Not Applicable	

Laboratory protocol	Information included in the manuscript?	In which section is the information available? (Reagents and Tools Table, Materials and Methods, Figures, Data Availability Section)
Provide DOI OR other citation details if **external detailed step-by-step protocols** are available.	Yes	Methods

Experimental study design and statistics	Information included in the manuscript?	In which section is the information available? (Reagents and Tools Table, Materials and Methods, Figures, Data Availability Section)
Include a statement about **sample size** estimate even if no statistical methods were used.	Yes	Figure legends
Were any steps taken to minimize the effects of subjective bias when allocating animals/samples to treatment (e.g. **randomization procedure**)? If yes, have they been described?	Not Applicable	
Include a statement about **blinding** even if no blinding was done.	Not Applicable	
Describe **inclusion/exclusion criteria** if samples or animals were excluded from the analysis. Were the criteria pre-established?<br><br>If sample or data points were omitted from analysis, report if this was due to attrition or intentional exclusion and provide justification.	Not Applicable	
For every figure, are **statistical tests** justified as appropriate? Do the data meet the assumptions of the tests (e.g., normal distribution)? Describe any methods used to assess it. Is there an estimate of variation within each group of data? Is the variance similar between the groups that are being statistically compared?	Yes	Methods and figure legends

Sample definition and in-laboratory replication	Information included in the manuscript?	In which section is the information available? (Reagents and Tools Table, Materials and Methods, Figures, Data Availability Section)
In the figure legends: state number of times the experiment was **replicated** in laboratory.	Yes	Figure legends
In the figure legends: define whether data describe **technical or biological replicates**.	Yes	Figure legends

Ethics

Ethics	Information included in the manuscript?	In which section is the information available? (Reagents and Tools Table, Materials and Methods, Figures, Data Availability Section)
Studies involving **human participants**: State details of **authority granting ethics approval** (IRB or equivalent committee(s), provide reference number for approval.	Not Applicable	
Studies involving **human participants**: Include a statement confirming that **informed consent** was obtained from all subjects and that the experiments conformed to the principles set out in the WMA Declaration of Helsinki and the Department of Health and Human Services Belmont Report.	Not Applicable	
Studies involving **human participants:** For publication of **patient photos**, include a statement confirming that consent to publish was obtained.	Not Applicable	
Studies involving experimental **animals**: State details of **authority granting ethics approval** (IRB or equivalent committee(s), provide reference number for approval. Include a statement of compliance with ethical regulations.	Not Applicable	
Studies involving **specimen and field samples:** State if relevant **permits** obtained, provide details of authority approving study; if none were required, explain why.	Not Applicable	

Dual Use Research of Concern (DURC)	Information included in the manuscript?	In which section is the information available? (Reagents and Tools Table, Materials and Methods, Figures, Data Availability Section)
Could your study fall under dual use research restrictions? Please check biosecurity documents and list of **select agents and toxins** (CDC): https://www.selectagents.gov/sat/list.htm	Not Applicable	
If you used a select agent, is the security level of the lab appropriate and reported in the manuscript?	Yes	Methods
If a study is subject to dual use research of concern regulations, is the name of the **authority granting approval and reference number** for the regulatory approval provided in the manuscript?	Yes	Methods

Reporting

The MDAR framework recommends adoption of discipline-specific guidelines, established and endorsed through community initiatives. Journals have their own policy about requiring specific guidelines and recommendations to complement MDAR.

Adherence to community standards	Information included in the manuscript?	In which section is the information available? (Reagents and Tools Table, Materials and Methods, Figures, Data Availability Section)
State if relevant guidelines or checklists (e.g., **ICMJE, MIBBI, ARRIVE, PRISMA**) have been followed or provided.	Not Applicable	
For **tumor marker prognostic studies,** we recommend that you follow the **REMARK** reporting guidelines (see link list at top right). See author guidelines, under 'Reporting Guidelines'. Please confirm you have followed these guidelines.	Not Applicable	
For **phase II and III randomized controlled trials**, please refer to the **CONSORT** flow diagram (see link list at top right) and submit the CONSORT checklist (see link list at top right) with your submission. See author guidelines, under 'Reporting Guidelines'. Please confirm you have submitted this list.	Not Applicable	

Data Availability

Data availability	Information included in the manuscript?	In which section is the information available? (Reagents and Tools Table, Materials and Methods, Figures, Data Availability Section)
Have **primary datasets** been deposited according to the journal's guidelines (see 'Data Deposition' section) and the respective accession numbers provided in the Data Availability Section?	Yes	Data availability
Were **human clinical and genomic datasets** deposited in a public access-controlled repository in accordance to ethical obligations to the patients and to the applicable consent agreement?	Not Applicable	
Are **computational models** that are central and integral to a study available without restrictions in a machine-readable form? Were the relevant accession numbers or links provided?	Not Applicable	
If publicly available data were reused, provide the respective **data citations in the reference list**.	Yes	References
