# Supplementary material for: E4BP4 safeguards brown fat mitochondria from obesity-induced fragmentation via ceramide repression
Source: EMBO Rep. 2026 Jun 22;27(15):4376–411. doi: 10.1038/s44319-026-00826-0 (PMC13458363; doi:10.1038/s44319-026-00826-0)
Supplement: Supplementary file 16 [file 44319_2026_826_MOESM7_ESM.zip › Movie EV1/Legend.docx]

Movie EV1. Live-cell fluorescence of MitoDendra2 in primary brown adipocytes

(related to Fig. 3G).
